# The flow of anisotropic nanoparticles in solution and in blood

**DOI:** 10.1002/EXP.20220075

**Published:** 2023-10-10

**Authors:** Jordan Thomas Lovegrove, Ben Kent, Stephan Förster, Christopher J. Garvey, Martina H. Stenzel

**Affiliations:** ^1^ Centre for Advanced Macromolecular Design School of Chemistry The University of New South Wales Sydney New South Wales Australia; ^2^ Forschungszentrum Jülich GmbH Jülich Germany; ^3^ Forschungsneutronenquelle Heinz Maier‐Leibnitz FRM II and Physik Department E13 Technische Universität München Garching Germany

**Keywords:** anisotropic nanoparticles, blood, drug delivery, microfluidic, microscopy, small angle X‐ray scattering

## Abstract

The alignment of anisotropic nanoparticles in flow has been used for a range of applications such as the preparation of strong fibres and the assembly of in‐plane aligned 1D‐nanoobjects that are used for electronic devices, sensors, energy and biological application. Important is also the flow behaviour of nanoparticles that were designed for nanomedical applications such as drug delivery. It is widely observed that non‐spherical nanoparticles have longer circulation times and a more favourable biodistribution. To be able to understand this behaviour, researchers have turned to analyzing the flow of non‐spherical nanoparticles in the blood stream. In this review, an overview of microfluidic techniques that are used to monitor the alignment of anisotropic nanoparticles in solution will be provided, which includes analysis by small angle X‐ray scattering (SAXS) and polarized light microscopy. The flow of these nanoparticles in blood is then discussed as the presence of red blood cells causes margination of some nanoparticles. Using fluorescence microscopy, the extent of margination can be identified, which coincides with the ability of nanoparticles to adhere to the cells grown along the wall. While these studies are mainly carried out in vitro using blood, initial investigations in vivo were able to confirm the unusual flow of anisotropic nanoparticles.

## Graphical abstract

There is an increased interest in the use of non‐spherical nanoparticles for drug delivery as they often display better blood circulation time. However, there is little understanding of the behaviour of these nanoparticles in blood flow. Microfluidic devices can now gain better insight as alignment of anisotropic nanoparticles as well as their margination towards the blood vessel wall can be directly observed .

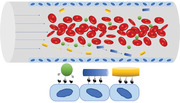

## 1 INTRODUCTION

The efficacy of nanoparticles as drug delivery vehicles depends on their ability to deliver a therapeutic payload to the desired targets before the nanoparticle is cleared from the body. This is a long journey as nanoparticles need to overcome a series of obstacles until they reach their final destination, which is usually inside the cell: Once nanoparticles enter the blood stream, they are typically covered with a layer of proteins. This protein corona then determines the fate of the nanoparticle.^[^ ^1^ ^]^ Initially, renal clearance and removal by the mononuclear phagocytic system (MPS) will eliminate a large amount of nanoparticles and redirect them for example to liver and spleen. Even if nanoparticles reach their target such as a solid tumour, extravasation and subsequent penetration into the tumour can be challenging. Finally, the nanoparticles need to be taken up by cells and the drug released to deliver the active compound. All these steps are dependent on the properties of the nanoparticles.^[^ ^2^ ^]^

Size and shape are important considerations when designing particles to overcome systematic barriers in the body such as filtration, macrophage clearance and cell entry.^[^ ^3^ ^]^ Spherical nano‐carriers dominate the literature, mainly due to their ease of synthesis and characterization. However, in nature many pathogenic viruses, bacteria and fungi are found in a variety of shapes such as filamentous, oblate or rod shapes.^[^ ^4^ ^]^ Bacteria capable of morphological changes, commonly transform from a spherical to filamentous shape, with this change reducing phagocytosis from the host by minimizing surface area in contact with the phagocyte.^[^ ^5^ ^]^

This inspired numerous studies that explored the correlation between the efficiency of non‐spherical and spherical particles in the context of drug delivery, however drawing simple conclusions about what are the most important design parameters to consider becomes difficult.^[^ ^6^ ^]^ There is a strong focus in literature on trying to understand the relationship between shape and cellular uptake. Influence of particle size and shape on cell‐uptake is dependent on what cell‐line is chosen, where no clear preferences for spherical nanoparticles can be seen.^[^ ^7^ ^]^ Similarly, shape has a profound impact on particle internalization and distribution. For instance, oblates and rods were observed to have lower internalization rate in human mesenchymal stem cells when compared to spherical particles.^[^ ^8^ ^]^ However other studies indicate that particles with higher aspect ratios translocate more readily into cells than spherical particles of equivalent size, especially in tumour cells.^[^ ^9^ ^]^ This also applies to discoidal or platelet‐like nanoparticles that have higher cellular uptake^[^ ^10^ ^]^ and better tumour accumulation in mice.^[^ ^11^ ^]^ Despite this, no clear design principles can be extracted from the literature, with there being numerous studies indicating spherical particles enter cells better than non‐spherical particles as well as the opposite.^[^ ^12^ ^]^ Key factors such as cell‐line and particle aspect ratio become important for each individual study, making broad conclusions on particle shape and cell entry difficult to summarize.^[^ ^13^ ^]^

Cell uptake studies, however, concern themselves with the behaviour of nanoparticles in case they might reach the tumour cells. Nanoparticles are however subject to a long journey starting with circulation in the blood stream to tumour extravasation and tumour penetration before the nanoparticles finally reach their target.^[^ ^2^ ^]^ A recent analysis has shown that less than 1% of nanoparticles are delivered to the tumour.^[^ ^14^ ^]^ It appears therefore more crucial that we enhance our understanding on the behaviour of nanoparticles in the initial stages after administration. Animal models can provide an insight into the biodistribution. Numerous studies have shown that non‐spherical nanoparticles have a favourable biodistribution as longer rod‐shaped nanoparticles were observed to have longer circulation time in the blood although rigidity was found to play an important role. It was proposed that the alignment of anisotropic nanoparticles in flow reduces phagocytosis, with anisotropic particles peeling off phagocytes they encounter, which leads ultimately to increased residence time in the body.^[^ ^15^ ^]^ The reader is referred to review articles that discuss the behaviour of non‐spherical nanoparticles in vitro and in vivo.^[^ ^15^, ^16^ ^]^

**FIGURE 1:**
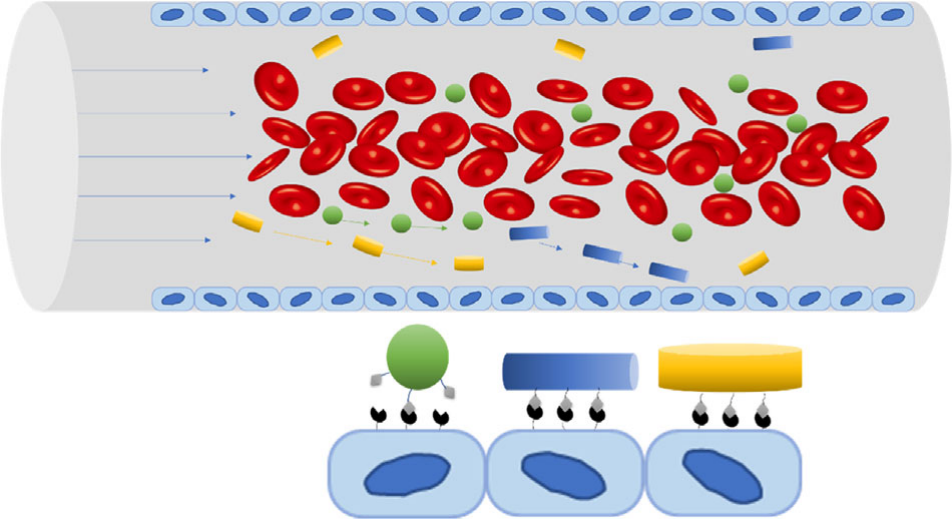
Margination of nanoparticles in the blood stream. (A) Margination of spherical, rod‐like and discoidal particles in the blood and (B) adhesion of particles to endothelial wall is dependent on surface area.

More in‐depth studies on the fate of these nanoparticles revealed that non‐spherical nanoparticles can have a tendency to marginate to the wall of the blood vessel where some particle can adhere to the endothelial cell (Figure 1).^[^ ^17^ ^]^ For example, in vivo evaluation of spherical and rod‐shaped microparticles has shown that rods target and bind to inflamed endothelium in the aortae of mice to a larger extent than spheres.^[^ ^18^ ^]^ This behaviour will have implication on the biodistribution of nanoparticles and circulation time. However, there is little focus so far in the literature on the flow of nanoparticles in the blood stream although it might provide the key to occurring phagocytosis and extravasation. The behaviour of nanoparticles in the blood stream is not only important to understand the fate in tumours, but their alignment and orientation will also affect interaction with blood cells^[^ ^19^ ^]^ and their interparticle collision,^[^ ^20^ ^]^ which is an important factor when either preventing uptake by blood cells or enhancing it such in the case of nanoparticle‐based vaccine delivery.^[^ ^21^ ^]^

In this review, the behaviour of non‐spherical particles in flow will be examined, including current methods for microfluidic device fabrication and characterization techniques suitable to understanding the orientation and alignment of anisotropic particles in flow.

## 2 MICROFLUIDICS TO STUDY ANISOTROPIC PARTICLES

### 2.1 Microfluidic devices as blood vessel analogues

An important aspect of particle design, besides particle shape and size, commonly overlooked is the behaviour of particles in flow. The alignment, orientation and fluid dynamics of particles is directly linked to particle shape.^[^ ^22^ ^]^ Evaluating the orientation of particles in flow will help to predict crucial behaviour of particles, such as which axis of symmetry aligns with flow direction, tumbling and rotational modes and the time scale of Jeffery orbits.^[^ ^22^, ^23^ ^]^ These properties can help understand the behaviour of particles in the bloodstream such as margination and adhesion, which are essential factors for the effectiveness of a drug delivery vehicle.

**FIGURE 2:**
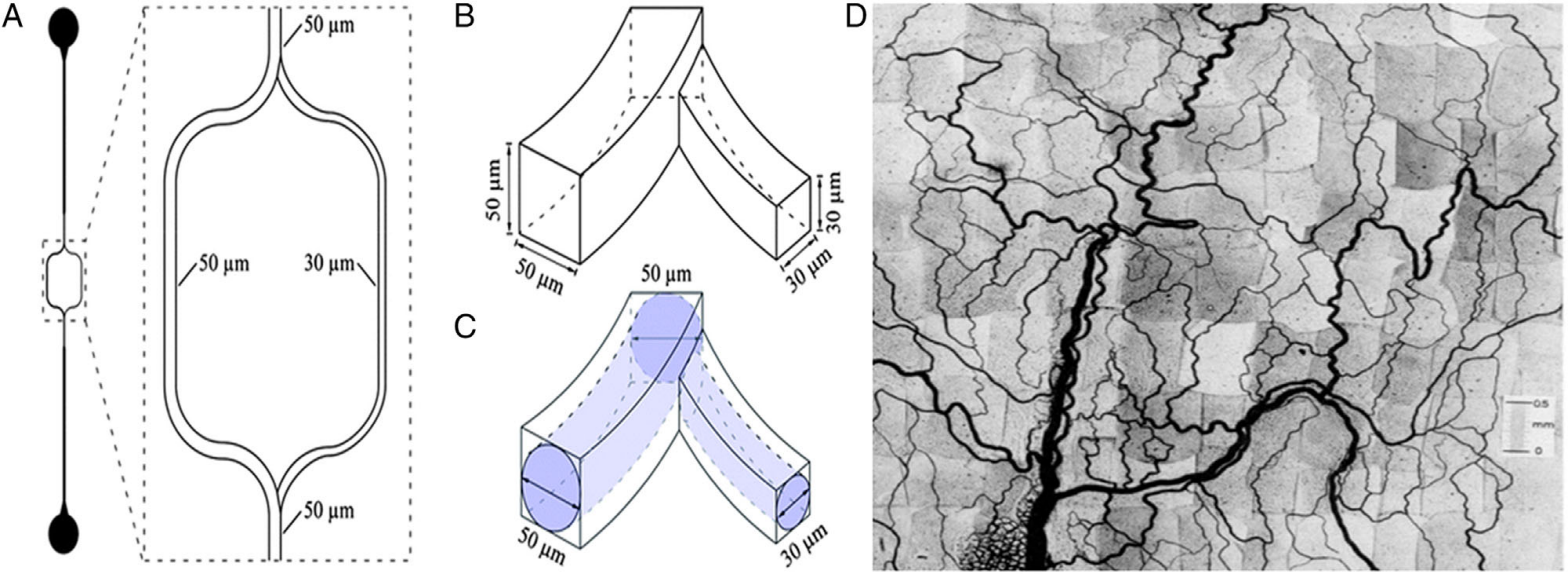
Comparison between (D) complex mesenteric microvasculature network and a microfluidic device that is inspired by blood vessels (A); (A) microfluidic devices as a synthetic blood vessel analogue that bifurcates into a larger and a smaller microchannel; (B) square channels as obtained after lithography and (C) after modifying the square channel into round ones using PDMS and hot air. (A–C) Reproduced with permission.^[^ ^24d^ ^]^ Copyright 2011, Royal Society of Chemistry; (D) Reproduced with permission.^[^ ^24d,e^ ^]^ Copyright 1986, American Physiological Society.

Microfluidic devices present a way to study particle dynamics in micro confinement, providing a useful synthetic mimic for blood vessels. Blood vessels in the body range from 8 µm in capillaries to 5000 µm in arteries, these dimensions can be emulated using microfluidic devices, with precise size and geometrical control that mimic the complex architectures found in the body (Figure 2).^[^ ^24^ ^]^

Microfluidic devices can be fabricated reliably using photolithography.^[^ ^25^ ^]^ Using traditional microfluidic fabrication techniques, the microfluidic devices are generated to have a rectangular cross‐section, which can be modified to be circular (Figure 2).^[^ ^26^ ^]^ However, vessels in the microvasculature are rarely perfectly circular, with many irregular geometries observed. These devices however provide a useful starting point for studying particle orientation, alignment, margination and adhesion under physiologically relevant conditions.^[^ ^24d^ ^]^

### 2.2 Fabrication of microfluidic devices

Fabricating microfluidic devices using photolithography is usually achieved by spin coating a silicon wafer with a polymeric photoresist, commonly a derivative of SU‐8 (Bisphenol A Novolac epoxy with eight epoxy groups). The photoresist layer is then immediately cured with the desired channel design using light. Once this master device is made, polydimethylsiloxane (PDMS) pre polymer is mixed with a curing agent at a ratio of 10:1 and poured onto the master silicon wafer and cured for several hours at 70°C. After the PDMS is cured, it is removed from the silicon wafer and inlet and outlet holes are punched into the device. Plasma bonding is then used to bond the PDMS device onto a surface, commonly a glass microscopy slide.^[^ ^25b^ ^]^

**FIGURE 3:**
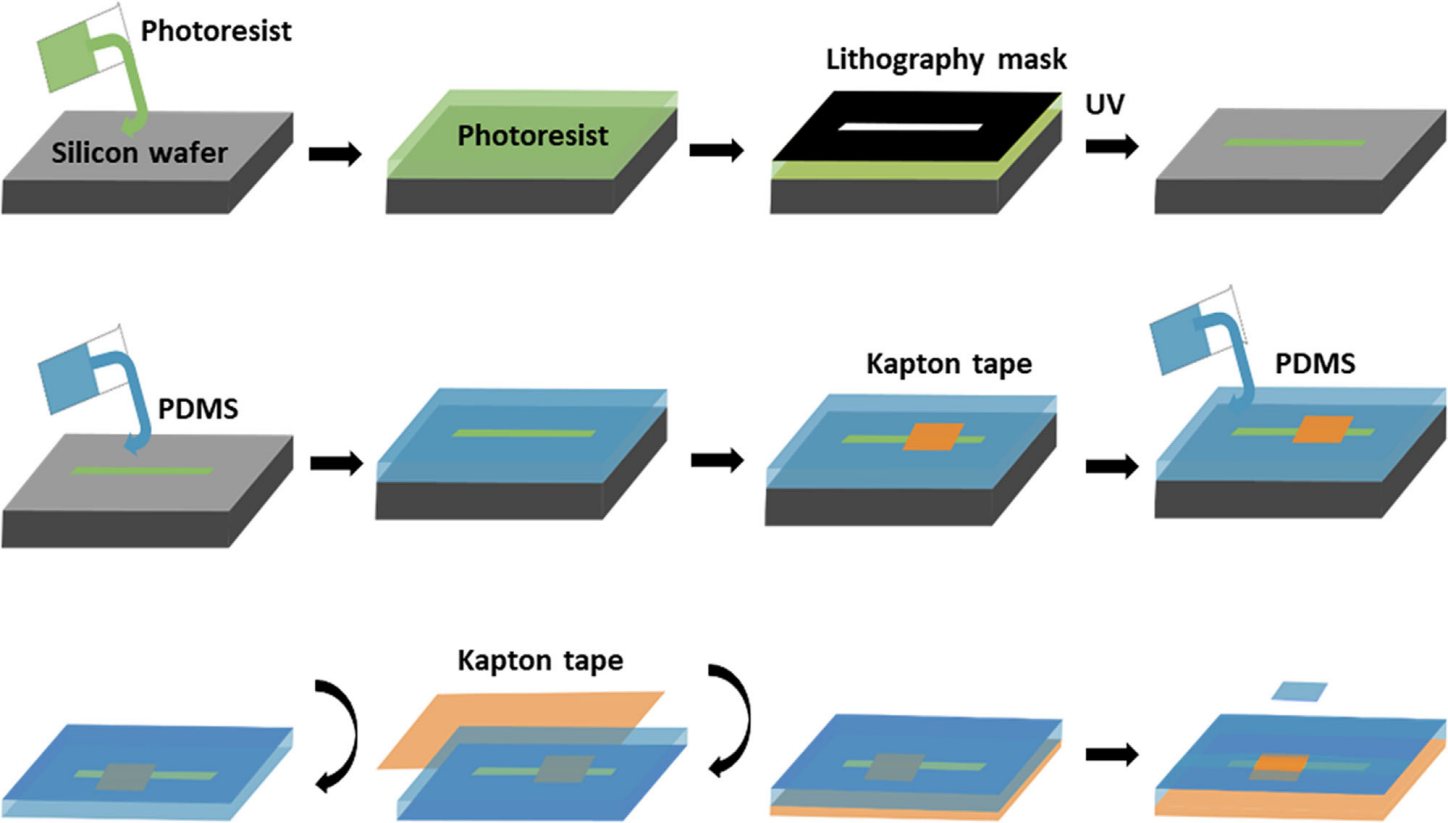
Workflow for the preparation of microfluidic channels. Fabrication of microfluidic devices optimized for acquisition of spatially resolved X‐ray scattering based on poly dimethyl sulfoxide (PDMS) lithography and Kapton windows.

One limitation of traditional PDMS microfluidic devices is that they rely solely on traditional microscopy techniques for in situ analysis. By combining microfluidic devices with SAXS, averaged particle information such as alignment, orientation, and particles concentration at various points across the channel can be accurately determined over the volume illuminated by the X‐ray beam. However traditional PDMS microfluidic devices are quite limiting for SAXS measurements, with the PDMS and glass causing an inconsistent and high background signal, which limits useful structural information, from in particular, weak SAXS signals. A modification to the traditional microfluidic fabrication process was devised by Evans et al., whereby a Kapton tape window is added to the device, enabling consistent background subtraction.^[^ ^27^ ^]^ This process is outlined in Figure 3.

The deviation from the traditional method occurs when PDMS is poured onto the master device, with a small amount poured to cover the master device up to the height of the microchannels, with excess uncured PDMS removed. This thin layer of PDMS is then cured and a layer of Kapton tape is placed directly onto the silicon wafer. More PDMS is then poured over the silicon wafer with the Kapton tape and cured. Finally, the device is removed and Kapton tape is added along the microfluidic device directly in contact with the silicon wafer. With a small hole cut through the cured PDMS to expose the Kapton tape, a microfluidic device is made with the area of interest being sealed solely with Kapton tape, which provides a very consistent background X‐ray scattering signal. Furthermore, the rectangular cross‐section of the device is suited to the subtraction of a consistent background signal. With devices able to measure particles in a variety of environments, extracting valuable information about the behaviour of particles in flow now becomes possible.

**FIGURE 4:**
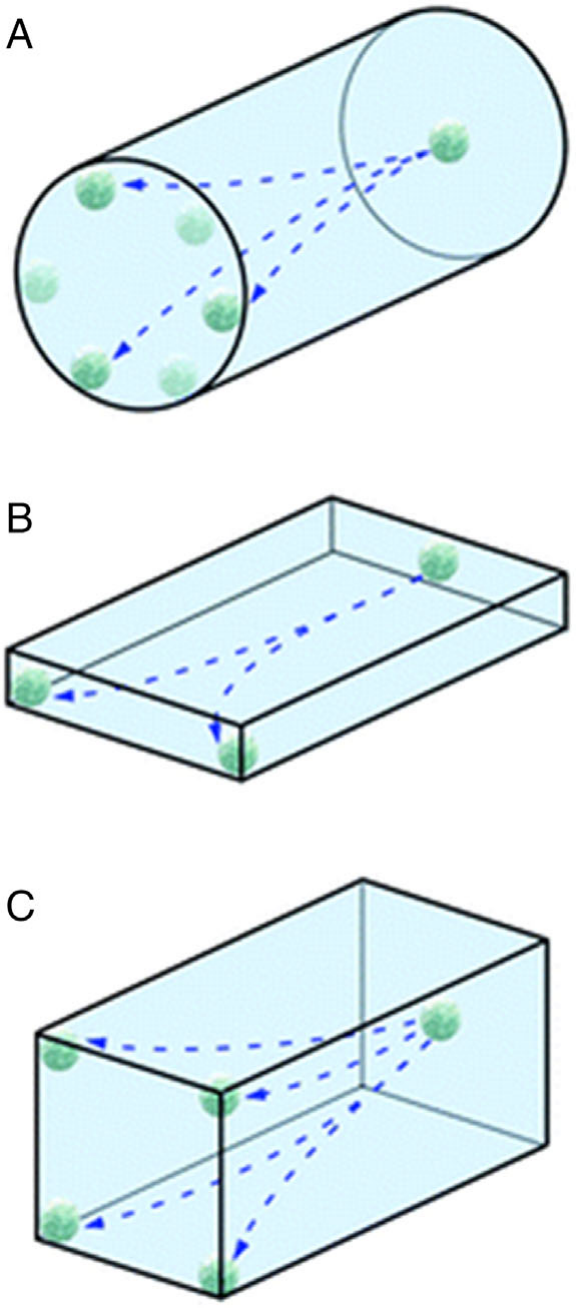
Pathways of particles in channels. Possible margination paths for leukocytes flowing with whole blood in (A) circular channel, (B) high aspect ratio rectangular channel and (C) low aspect‐ratio rectangular channel. Reproduced with permission.^[^ ^24d^ ^]^ Copyright 2011, Royal Society of Chemistry.

Microfluidic channel design influences the flow of nanoparticles.^[^ ^28^ ^]^ Importantly, laminar flow can be generated inside these devices with PDMS based microfluidic devices being widely used to study hemodynamic, particle alignment and particle adhesion.^[^ ^17^, ^28^, ^29^ ^]^ Important factors to consider when using microfluidic devices to observe margination is channel shape and size. In a paper by Yang et al., a theoretical prediction on the deviation of leukocyte margination to the channel wall was made based on the cross‐sectional shape of the microfluidic channel used, as depicted in Figure 4.^[^ ^24d^ ^]^

Circular cross sections do not influence the margination profile of the particle in flow, with equiprobable location of the particle found along the perimeter of the channel. In rectangular channels with a high aspect ratio, particles will preferentially accumulate at the sidewalls. While in lower‐aspect ratio channels that are near in shape to square, particle accumulation will be found higher in the corners, with the choice of microfluidic device channel influencing measurement outcomes.

**FIGURE 5:**
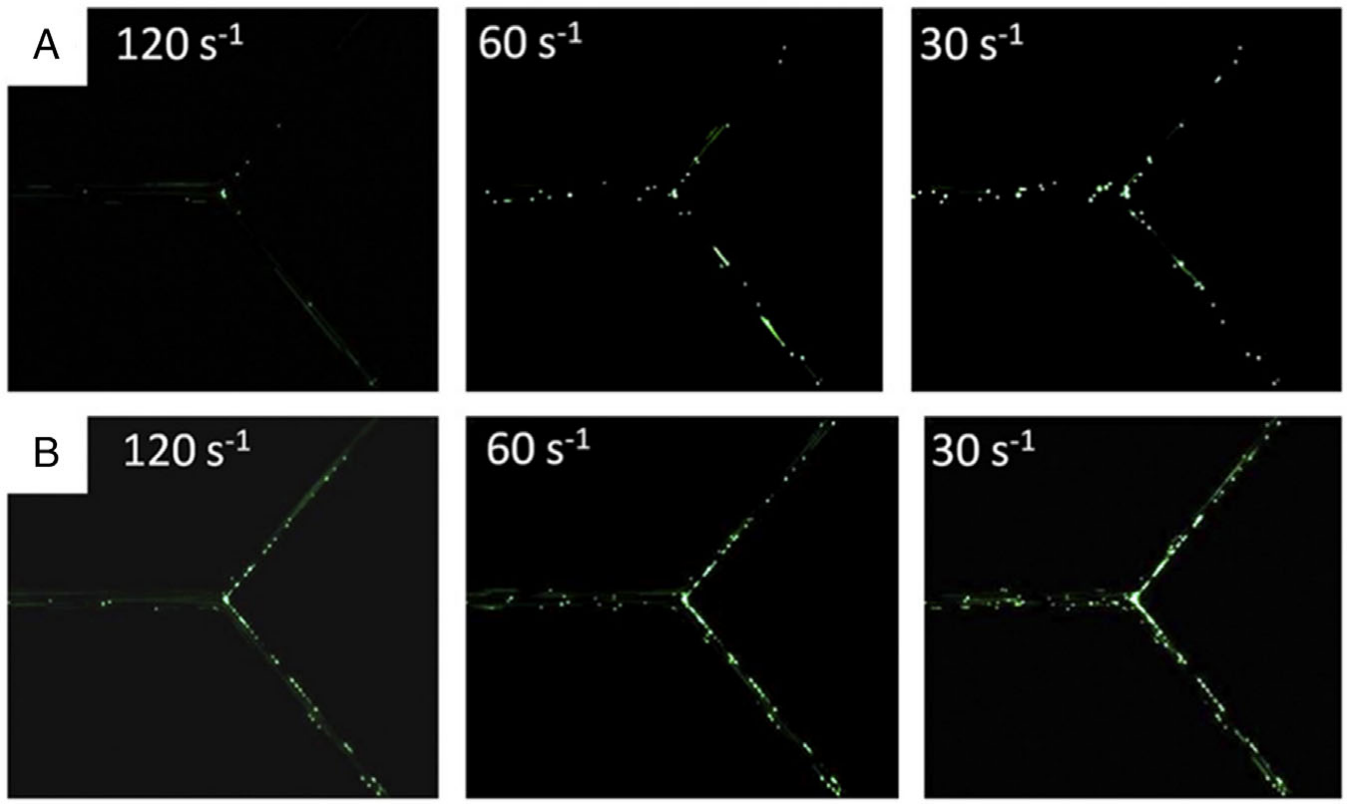
Adhesion of nanoparticles on BSA‐coated channels. Fabrication of microfluidic device with bifurcation coated with BSA on channel walls to study the adhesion of anti‐BSA coated (A) spheres or (B) elliptical disks at various shear rates. Reproduced with permission.^[^ ^28c^ ^]^ Copyright 2010, Elsevier.

More complex structures that closely resemble the microvasculature can be achieved by adding a bifurcation (branching) to a linear channel, to mimic the branching nature of blood vessels. Doshi et al. prepared microchannels with a bifurcation angle of 90° using standard photolithography processes (Figure 5) with the addition of coating the channel walls with a bovine serum albumin (BSA) solution.^[^ ^28c^ ^]^

The bifurcation when combined with channel coating, allows for a simple way to emulate the endothelial wall in blood vessels and measure the adhesion of particles to the channel wall under flow conditions.

However, the previously described channels all have rectangular cross‐sections. Fabricating spherical channels have advantages over rectangular channels such as predictable shear flow. These channels have been fabricated using several methods, such as removal of a wire embedded into PDMS and flowing air through a channel with uncrosslinked PDMS.^[^ ^26^, ^30^ ^]^ A simple way spherical channels can also be generated is by the removal of solder wire embedded into PDMS. Solder wire with a melting point of 190°C is inserted into uncured PDMS and, due to the flexibility of the wire, several complex channel shapes can be generated such as helixes and curved structures.^[^ ^30^ ^]^ The PDMS device with embedded solder wire is then heated to 190°C to melt the wire, which can then be expelled from the device with no damage to the structure.^[^ ^30^ ^]^ Non‐PDMS devices, mainly commercially available glass and silica capillaries, are also commonly used to study particle flow, providing a simple and cheap way to mimic blood vessels, with the downside of limited customizability.^[^ ^29^ ^]^

### 2.3 Organ on a chip devices

**FIGURE 6:**
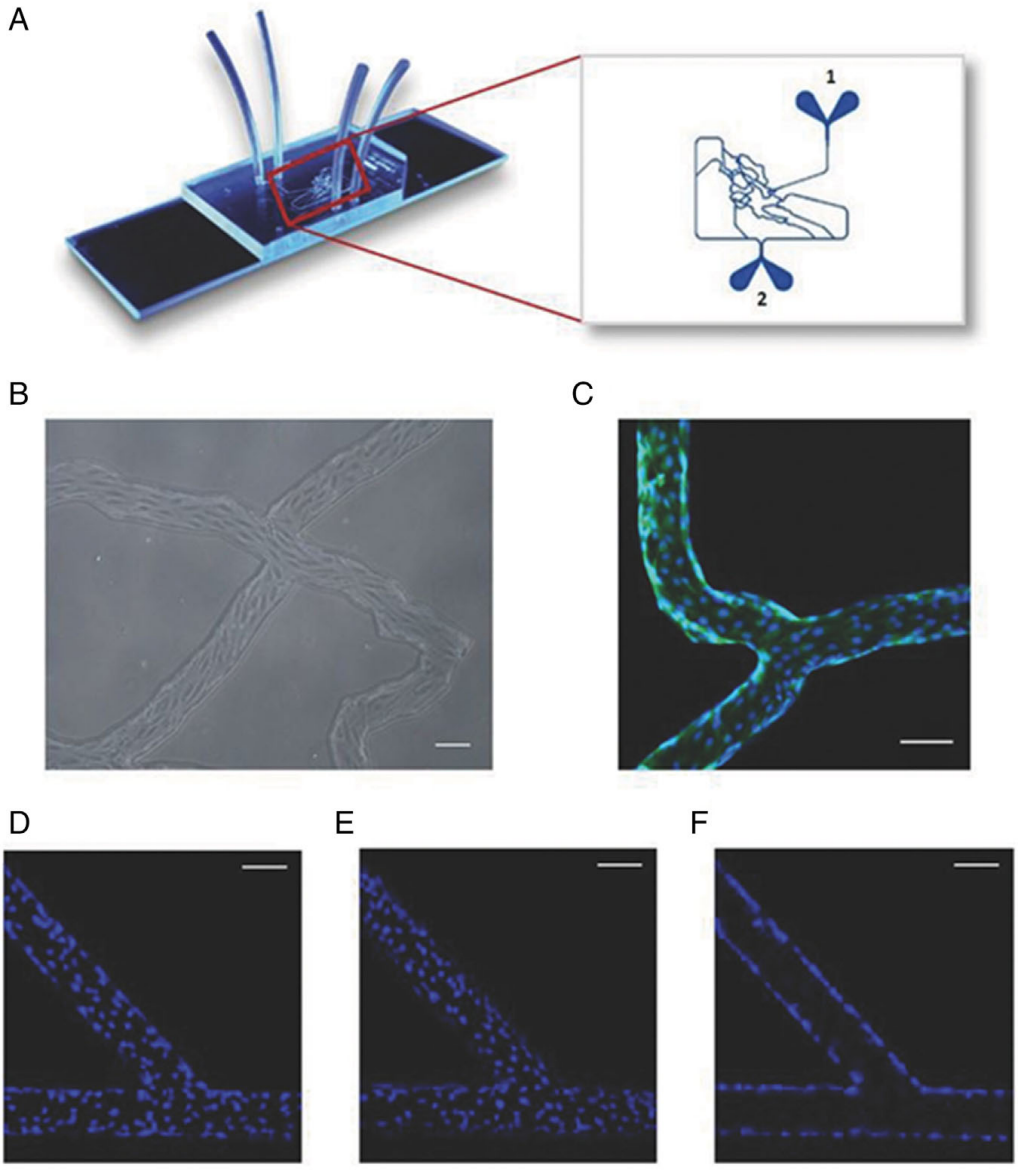
Adhesion of nanoparticles on HUVEC coated microfluidic channels. (A) Model synthetic microvascular network: (1) inlet port where cell growth media with or without nanoparticles is injected into the system and (2) outlet port where perfusate from the system is collected. Adapted with permission from SynVivo, Inc., https://www.synvivobio.com/product/microvascular‐network‐chip‐smn1‐d001/ (accessed: March 2023). (B) Bright field and (C) confocal images of a confluent layer of HUVECs in flow channels. Cells were stained with Calcein AM (cytoplasm: green channel) and Hoechst 33342 (nucleus: blue channel). Nucleus of HUVECs stained with Hoechst 33342 was imaged (D) at the bottom, (E) at the top, and (F) on the sides of the vascular pipeline. Scale bars = 100 µm. Reproduced with permission.^[^ ^31^ ^]^ Copyright 2018, Wiley.

More complex microfluidic devices, that can emulate the cellular structure of several organs have also recently been synthesized by combining microfluidic device fabrication with cell culturing techniques. An example of this process is outlined by Hasan et al., where blood vessel analogues were made.^[^ ^28a^ ^]^ This multistep process involves placing a small glass capillary (1.2 mm internal diameter) into a PDMS matrix to create a straight channel. After several preparation steps, this channel is seeded with three layers of cells that closely resemble blood vessel structure, with fibroblasts, smooth muscle cells, and endothelial cells systematically inserted into the capillary.^[^ ^28a^ ^]^ Alternatively, commercial chips are available that can mimic blood vessels with their bends, curves and bifurcation more closely.^[^ ^31^ ^]^ These channels are then coated with Matrigel and can then be seeded for example with HUVECs as shown in Figure 6 to study the internalization of micelles under flow and static conditions.

In a recent review by Anh et al.,^[^ ^32^ ^]^ several distinct organ on a chip devices that have been fabricated are explored in relation to nanoparticle drug delivery, where lung, angiogenic blood vessels, breast cancer, and liver environments are generated.^[^ ^33^ ^]^ These devices add to the arsenal of techniques that can be used to test the efficacy of therapeutics while replicating complex in vivo conditions of the human body in vitro.

## 3 ANALYZING PARTICLES IN FLOW USING MICROFLUIDICS

### 3.1 Alignment of anisotropic particles in flow

Anisotropic particles demonstrate interesting properties under flow conditions, such as changes in orientation and alignment travelling through constrictions and bends.^[^ ^27^, ^34^ ^]^ While understanding the alignment of particles under flow conditions is often overlooked, integrating techniques to characterize alignment such as SAXS or polarized microscopy into particle characterization can help predict the fate of particles in vivo.

Particles alignment in flow can vary from complete disorder—particles are arranged in a random orientation with no preferred direction—to complete alignment where all particles are orientated in the same direction. Typically, alignment of particles will fall somewhere between these two extremes. The degree of alignment can vary spatially and temporally within the flow volume, adding complexity to the study of aligned non‐spherical particles.

This section discusses two measures of quantifying anisotropic particle alignment in flow—the orientation distribution function and the order parameter. Two experimental techniques to obtain these quantities are explained—SAXS and polarized light microscopy. Although fluorescent microscopy is the technique of choice when working with complex solutions such as nanoparticles in blood, this technique is not used to evaluate alignment but to identify the location of nanoparticles within channels.

**FIGURE 7:**
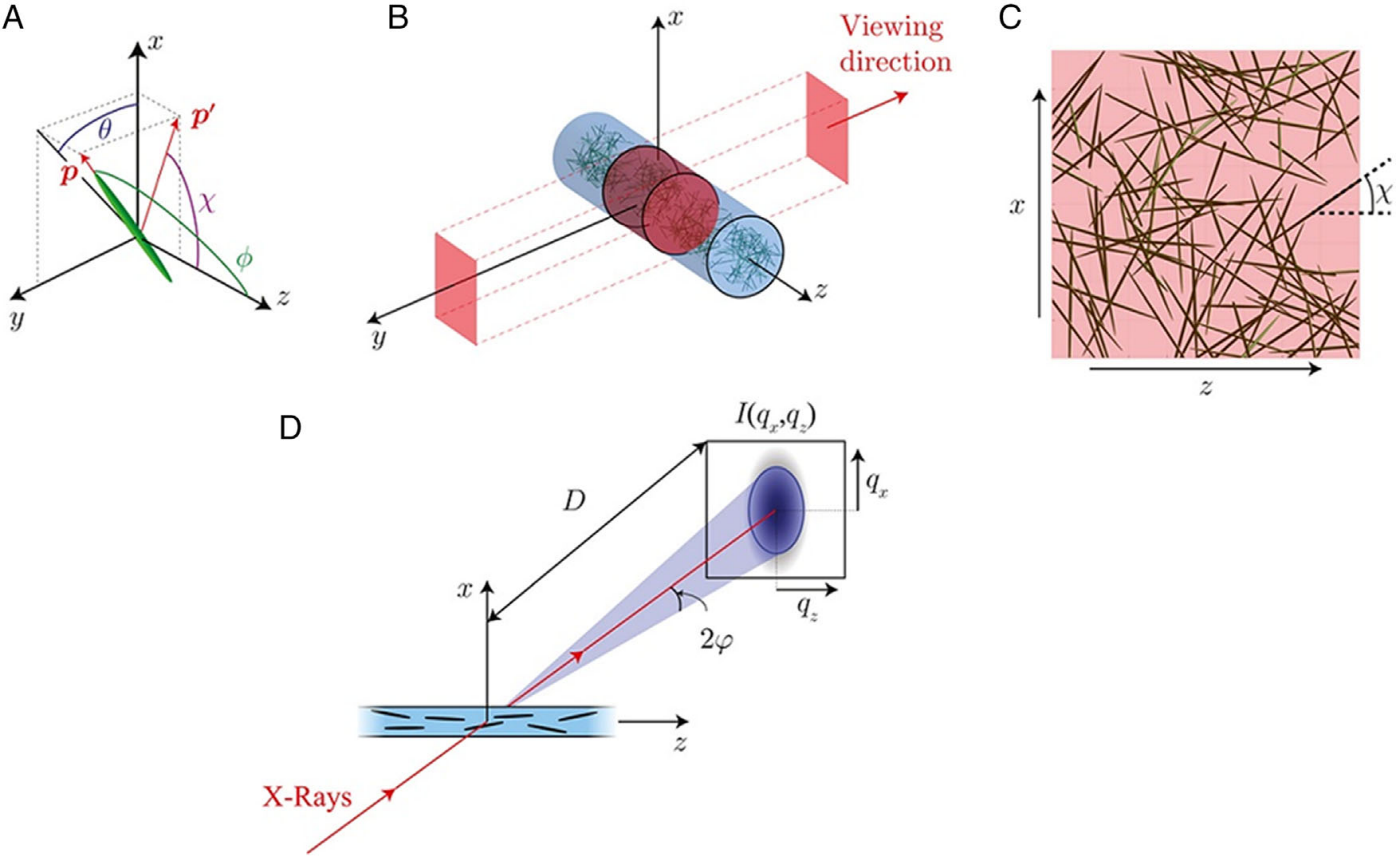
Definition of orientation of a non‐spherical particle. (A) Angle *ϕ* is the polar angle, the angle between the direction of flow and the major axis of the particle, angle *θ* is the angle between the major axis and the direction perpendicular to the flow. The projected angle χ is the angle of the major axis projected on to the *xz* plane—that is the viewing plane. (B) Projection shown in (C) in relation to the viewing direction; (D) scattering patterns in relation to the flow of nanoparticles and X‐ray direction. Reproduced with permission.^[^ ^35^ ^]^ Copyright 2018, American Chemical Society.

The first step in determining alignment is to define orientation. Typically, this is done by measuring angles of the particles with respect to the direction of flow. Non‐spherical particles are defined by a major axis and a minor axis. A simple example is to imagine an ellipsoid object flowing through a channel on a horizontal microscope slide. The major axis is the longest diameter that runs through the centre, while the minor axis is the shortest, and intersects at a right angle with the major axis. In a flow system with a defined direction of flow, we can describe their orientation with two angles—the polar angle *ϕ* and the azimuthal angle *θ* (Figure 7a). The polar angle is the angle between the major axis and the direction of flow, and the azimuthal angle is the angle between the major axis and the direction perpendicular to the flow. Thus, ellipsoidal particles with long axis aligned along the direction of flow have a polar angle of 0°. For all non‐zero polar angles, we also need to consider that the particle for any single polar angle could be orientated in a direction with respect to the direction perpendicular to the flow. Thus, the angle between the major axis and the direction perpendicular to the flow—the azimuthal angle *θ*—is also required to fully understand the particle's orientation.

Experimentally it is difficult to separate the polar and azimuthal angles. For example, if we were to view a particle along the axis perpendicular to the flow direction *z*, as is commonly the case experimentally, a particle with 0° azimuthal angle would give the same answer for all values of polar angle (Figure 7b). The particle is aligned along the direction of viewing, and the polar angle cannot be measured unless we change our viewpoint to view to particle from a different direction. Thus, the experimentally obtained value from measurements is typically the projected angle *χ*—the angle the particle projects to the viewer (Figure 7c). This is the case for both microscopy and for SAXS, which records a 2D scattering pattern for a 3D scattering volume (Figure 7d).

Deriving the polar and azimuthal angles from the experimentally obtained projected angle is non‐trivial and requires typically experimental modification.^[^ ^35^ ^]^ Rotation of the sample during measurement so that multiple viewpoints of the orientation are established, and the full 3D orientation can be derived, is the obvious method. However, this can be difficult for flow situations and requires complex analysis of many data sets. Vainio et al. proposed using a generalized normal distribution function to reconstruct a 3D view of the orientation, while Rosen et al. used a Legendre decomposition technique of the projected orientation to recover the 3D orientation. In many cases, it can be sufficient to use the projected angle information as a first approximation.^[^ ^35^, ^36^ ^]^

### 3.2 Orientation distribution function

The orientation distribution function (ODF) describes the alignment profile of particles within a volume. It plots the density of particles alignment on the vertical axis versus the projected angle on the horizontal axis. A system in which all particles are perfectly aligned in the direction of flow would show a delta function at 0° projected angle, while a completely unaligned system results in a flat ODF across all projected angles. In practice even in highly aligned systems there are particles orientated slightly off the 0° projected angle, with the particle numbers at a particular orientation decreasing the further away from 0° projected angle. Therefore, an aligned system would result in a peak (or peaks) with the amplitude and shape of the peak indicating the orientation order of the system.

**FIGURE 8:**
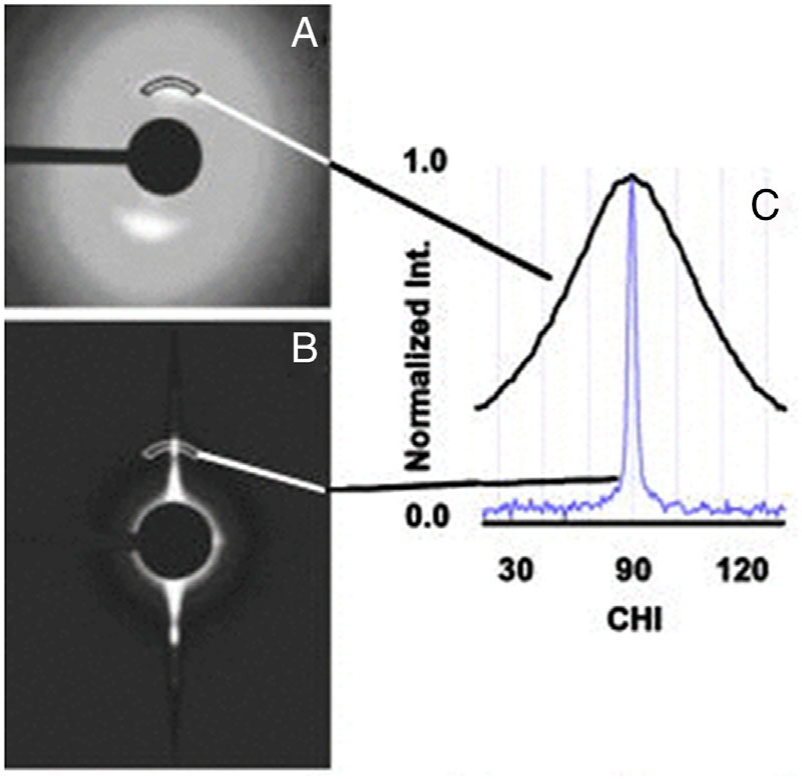
X‐ray analysis of actin in stationary solution and in flow. (A) 2D SAXS patterns of the protein F‐actin and (B) the same protein confined within a microchannel. The intensities as a function of azimuthal angle are shown in (C). Reproduced with permission.^[^ ^37^ ^]^ Copyright 2004, AIP Publishing.

The first step in understanding particle alignment using SAXS is to inspect the 2D SAS scattering patterns. Alignment can be seen by anisotropic scattering around the beam centre. The degree of anisotropy gives a first indication of the amount of orientation order of the particles (Figure 8). An example in point is the alignment of filamentous proteins F‐actin confined within microchannels.^[^ ^37^ ^]^ F‐actin is a long chain rod‐like biopolymer that forms supramolecular structures up to hundreds of nanometres. In quartz capillaries, partial alignment of the proteins leads to a broad rounded peak in the ODF, reflecting the many angles of particle orientation present in the system. When the proteins are confined with a microchannel, the restricted dimensions of the channel only allow the molecule to extend in one direction—with the rods aligned along the channel, regardless of the flow state. In this case the ODF shows a sharp peak centred on the projected angle of alignment of the system. SAXS patterns of F‐actin show some degree of anisotropy due to the nature of the proteins (Figure 8).

**FIGURE 9:**
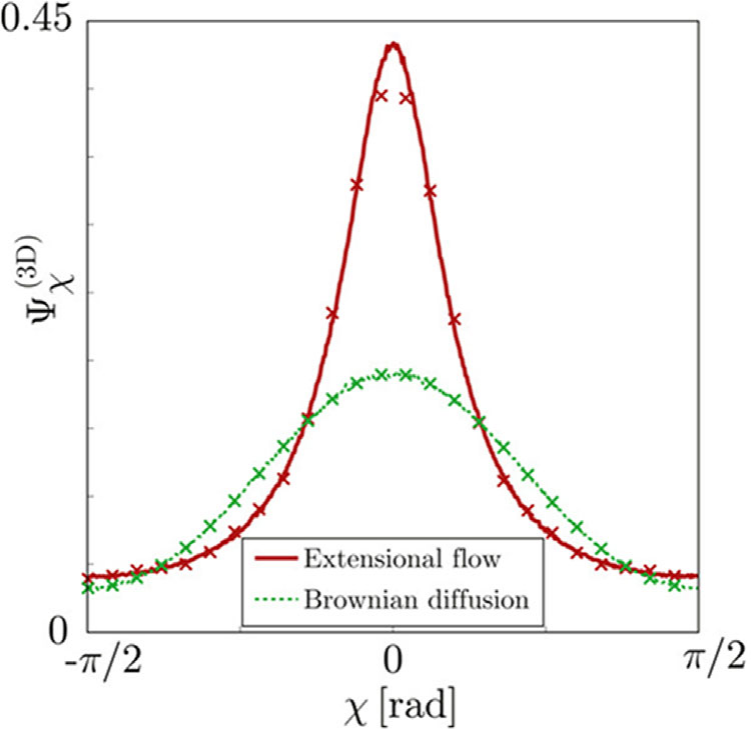
Order‐disorder function (OFD) of F‐actin in flow and in stationary solution. Comparison between ODFs Ψχ (3D) of F‐actin obtained with uniaxial extensional flow and Brownian rotary diffusion at approximately the same degree of alignment. Reproduced with permission.^[^ ^35^ ^]^ Copyright 2018, American Chemical Society.

In the case of flow induced alignment, the ODF takes on different shapes according to the orientational behaviour of particles in flow. Shape analysis of the ODF gives a first indication of the type of flow experienced by the particles. Shown in Figure 9 are two ODF's for different types of particle movement—extensional flow and Brownian diffusion.

Extensional flow, where the particles are pulled along the same streamline, results in a high well‐defined peak in the ODF, compared to the smoother lower curve for a system undergoing Brownian diffusion. Thus, analysis of the height and definition of peaks within the ODF gives a first indication of the type of flow. Rosen et al. demonstrated the shape of the ODF can be quantified by introducing a shape parameter *γ*, where:^[^ ^35^ ^]^

$$\gamma =\log\left(\frac{\log{\langle P_{4}\rangle }_{\chi }}{\log{\langle P_{2}\rangle }_{\chi }}\right)/\log\;2$$

The shape parameter is defined by a function composed of Legendre polynomials and gives a convenient threshold separating an extensional flow regime (*γ* ≤ 1) from Brownian diffusion (*γ* ≥ 1). This threshold can be used qualitatively or quantitively to understand the flow characteristics of the particles in the system.

### 3.3 Order parameter

The order parameter *S* provides a way to quantify the degree of alignment of particles. By integrating the area under the curve of an orientation distribution function, magnitude of the order parameter can be obtained:^[^ ^38^ ^]^

$$S=\int_{0}^{\pi }I\left(\theta \right)\left(\frac{3}{2}\cos^{2}\theta -\frac{1}{2}\right)\;\sin\theta \;d\theta$$

The order parameter should be normalized so that when the particles are isotopically orientated, the order parameter is 0, while for a perfectly aligned system, with all particles aligned along the direction of flow, the order parameter is 1:

$$\int_{0}^{\pi }I\left(\theta \right)\sin\theta \;d\theta =1$$

The order parameter is the mean of the second‐order Legendre polynomial *P*_2_, and complements the ODF by providing a single quantifying value of the degree of orientational order of the system.^[^ ^39^ ^]^

### 3.4 Techniques to measure alignment

#### 3.4.1 Small angle X‐ray scattering

**FIGURE 10:**
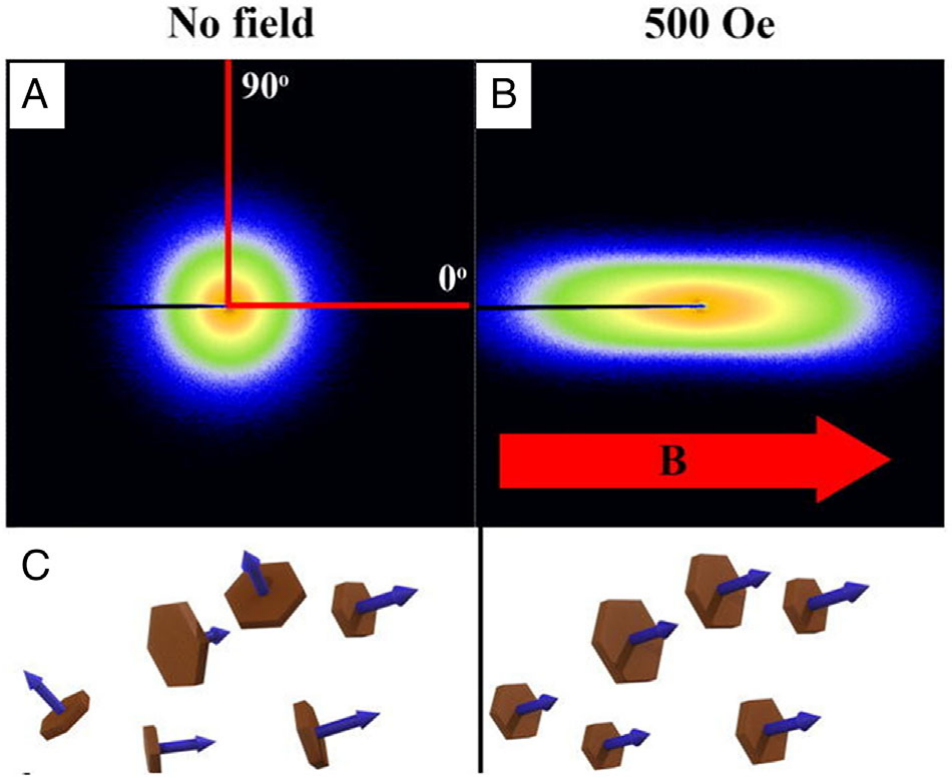
(A, B) Orientation and resulting SAXS pattern of nanoplatelets. SAXS patterns and (C) expected particle organization in zero field and at 500 Oe of magnetic hexaferrite nanoplatelets in water. Reproduced with permission.^[^ ^40^ ^]^ Copyright 2018, AIP Publishing.

Small angle X‐ray scattering (SAXS) can be used to resolve the spatial changes within a scattering volume. Samples with spatial correlations within the length scale of measurement will produce maxima in the far field on a SAXS detector. For a randomly orientated particle system these maxima have no preferred scattering direction and therefore display as rings around a centre beam on a 2D detector. The lack of preferred orientation of the particles means that SAXS intensity is constant around each ring.^[^ ^40^ ^]^ (Figure 10). The position of these rings from the beam is determined by the scattering vector *q*. Samples with orientation have preferred scattering directions resulting in intensity variation around the maxima rings. Thus, the first qualitative indication of alignment or orientation within the sample comes from inspecting the 2D SAXS detector image for variances of SAXS intensity as a function of azimuthal angle.^[^ ^40^ ^]^

The greater the variance of SAXS azimuthal intensity distribution (*I(q)*) the greater the alignment of particles in the scattering volume. This is observed by performing a radial average around set *q* value (*q* ± D*q*) containing a SAXS intensity ring of interest. Distinct from the azimuth angle defined earlier for non‐spherical particle alignment, the SAXS azimuthal angle is the angle from a chosen radius extending from the beam centre to a second radius also extending from the beam centre. An azimuthal average is performed by plotting the intensity of a ring within a chosen *q* range versus azimuthal angle. This can be observed in the example earlier of F‐actin (Figure 8). The azimuthal average plots intensity versus azimuthal angle from 0° to 360°. In the case of systems of particles, the SAXS patterns are mirrored, so that the intensity for two halves of the detector can be averaged and plotted versus azimuthal angle from 0 to 180°.

The q range within which to average intensities depends on the length scales of interest. Rosen et al. use *q* values corresponding to length scales between the minor and major dimensions in their study of cellulose nanofibrils.^[^ ^35^ ^]^ The azimuthal intensities within this range are averaged and the mean is taken.

The SAXS azimuthal average is proportional to the projected angle ODF of a system. This provides a first approximation of the orientation and alignment within a system and is often sufficient experimentally. To obtain a fully corrected ODF, corrections to the SAXS azimuthal average must be made for contributions due to isotropic scattering, and for the presence of particles that remain isotopically orientated regardless of the effects of flow. These corrections are found by obtaining an orientation distribution function from a highly aligned system in flow. For such a system, it is assumed that there are negligible particles orientated perpendicular to the flow. Thus, the scattering contribution for *χ* = 90° is solely due to the constant isotropic scattering, and not due to the aligned particles. This value is subtracted from all angles, so that the remaining intensity describes only the alignment of the particles.

**FIGURE 11:**
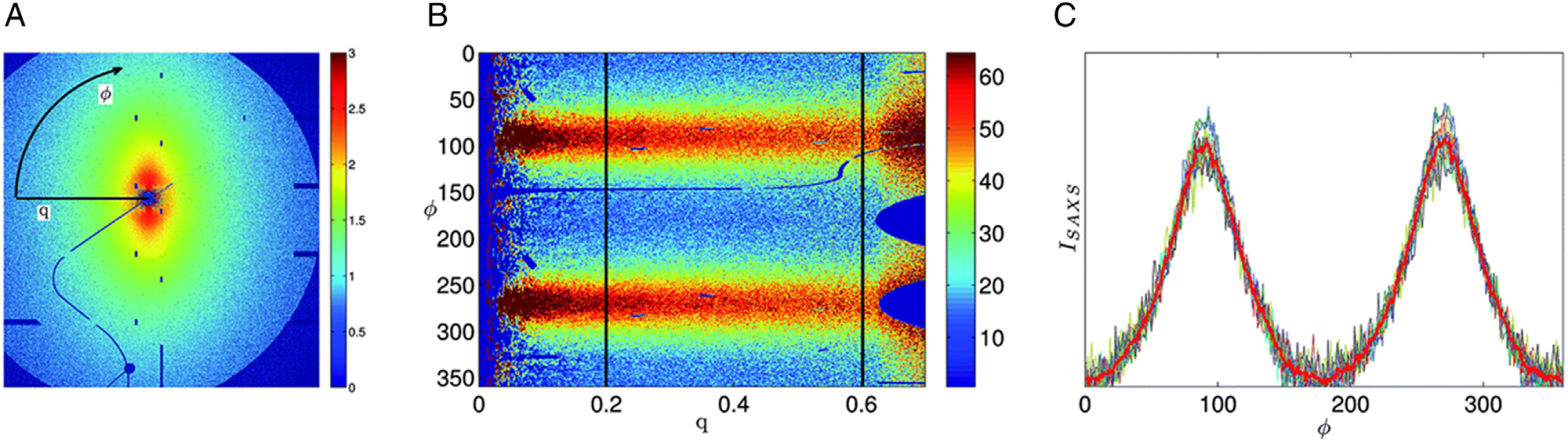
Orientation of nanoparticles as seen by SAXS analysis. (A, B) Transformation of 2D SAXS pattern from (C) Cartesian coordinates to azimuthal and radial coordinates. Orientation distribution functions and their mean in red for the *q* range shown with black vertical lines in (B). Reproduced with permission.^[^ ^34^ ^]^ Copyright 2015, Royal Society of Chemistry.

SAXS azimuthal intensity variation due to alignment can be visualized in different ways. In the case of Hakansson et al., they first perform an image transformation of the 2D SAXS pattern, with the red representing the most particle scattering from Cartesian coordinates to azimuthal and radial coordinates (Figure 11a,b).^[^ ^34^ ^]^ The transformed data (Figure 11b) is then discretized along the *x*‐axis into columns. Each column, representing a *q* value, is scaled to the other columns by the maximum intensity in that column. ODF's are then obtained for several values of *q*. The mean value of the functions is taken following verification they are similar within the chosen *q* range. The intensity is then presented as a function of azimuthal angle *θ* (Figure 11c).

#### 3.4.2 Polarized light microscopy to measure alignment

Polarized microscopy utilizes birefringence in materials to study alignment within a system. It is a complementary tool to SAXS in the study of ordered systems. SAXS provides local detailed orientation information through ODF's of a small scattering volume within a system, while polarized microscopy provides an overall magnitude of order within a system over a much larger volume.

Birefringence—the property of a single material having different refractive indices for different polarization directions—arises from highly ordered and aligned structures. The refractive index of a material determines the velocity at which a light ray travels through it. In birefringent materials, the two refractive indices lead to two different velocities of light passing through—a fast ray and slow ray. This means that the fast ray will have travelled a distance further beyond the boundary of the material by the time the slow ray reaches the boundary. This distance is known as retardation. As the two rays emerge from the material, they recombine to form a single ray. In the case of an isotropic material, there is no retardation, and the emerging ray is identical to the incident ray. When retardation is present, vector addition of the two rays leads to a new ray.

Polarization microscopy incorporates placing a polarizer before the specimen, and a second polarizer—the analyzer—after the specimen. These two polarizers have their polarization alignment set 90° apart. In the absence of retardation, incident light polarized by the first polarizer would not pass the second polarizer (the analyzer), resulting in a dark image. However, when retardation is present, as in the case of an ordered birefringent material, the new recombined wave has a component which will pass the analyzer, leading to an image brightness which depends on the retardation.

Polarized microscopy can be performed using a monochromatic or polychromatic white light source. In the former case, retardation due to birefringence leads to different brightness levels depending on the retardation. In the latter case, the resultant image due to retardation will show interference colours depending on the retardation.

##### 3.4.2.1 Relating birefringence signal to orientation

The square root of the birefringence intensity is related to the order parameter explored earlier where:^[^ ^41^ ^]^

$$B=\sqrt{I}\propto S_{\phi }=\left(\frac{3}{2}\cos^{2}\phi -\frac{1}{2}\right)$$

where *B* = relative birefringence, *I* = intensity, *S_φ_* = orientation parameter, *ϕ* = polar angle with respect to the flow direction.

*S_ϕ_* measures the relative alignment of the particles with respect to the flow direction. It is valid for dilute systems but not concentrated systems.^[^ ^39^ ^]^ Polarized light microscopy can be used to track the alignment of birefringent materials confined in micro channels, with changes in particles orientation seen by different refraction wavelengths, that can be visualized using a quarter wave plate.

### 3.5 Alignment of nanoparticles in flow

The alignment of anisotropic nanoparticles in flow is widely studied as it is important for a range of areas such as fibre spinning. In general, anisotropic nanoparticles align themselves in the direction of the flow, which is often used to generate aligned materials.^[^ ^42^ ^]^ This includes silver nanowires,^[^ ^43^ ^]^ clay platelets,^[^ ^44^ ^]^ tobacco mosaic virus,^[^ ^45^ ^]^ gold nanorods,^[^ ^45b^ ^]^ and bacteriophage M13,^[^ ^45b^ ^]^ cellulose nanofibrils,^[^ ^38^, ^46^ ^]^ gallium phosphide (GaP) nanowires,^[^ ^47^ ^]^ indium phosphide (InP) nanowires,^[^ ^47^ ^]^ and silicon (Si) nanowires.^[^ ^47^ ^]^ In general, alignment increases if the anisotropic nanoobjects has higher aspect ratios, the flow rate and concentrations are higher, the nanoparticle stiffness increased and the channels are narrower, although there is a lower channel size limit where the capillaries are easily blocked by the material. When the ratio of shear rate *γ* and rotation diffusion *D*_rot_ is much larger than 1, alignment will take place. Rotation diffusion *D*_rot_ is a function of the shape and size of the nanoparticle,^[^ ^48^ ^]^ while the shear rate increases in channels of smaller diameters and at higher volumetric flow rates. These observations are therefore not unexpected. In most cases the solutions with their aligned particles were either cast onto a substrate or the solution was processed into free‐standing fibers. After that the alignment was confirmed using microscopy techniques such as transmission electron microscopy. The alignment process in solution itself is often not directly observed and only the final aligned product is evaluated, and the fraction of ordered areas is determined. The quality of alignment during the process is often evaluated using birefringence, mostly as a qualitative tool to identify suitable concentrations and flow rates.^[^ ^44^, ^49^ ^]^ Occasionally the process is evaluated in more details and could often uncover surprising behaviours. Cylindrical block copolymer micelles were observed to orient themselves parallel to the channel walls as expected. However, after being pushed through a constriction, they start arranging themselves perpendicular to the flow in the centre of the channel while the parallel alignment was still in place closer to the wall.^[^ ^27b^ ^]^

**FIGURE 12:**
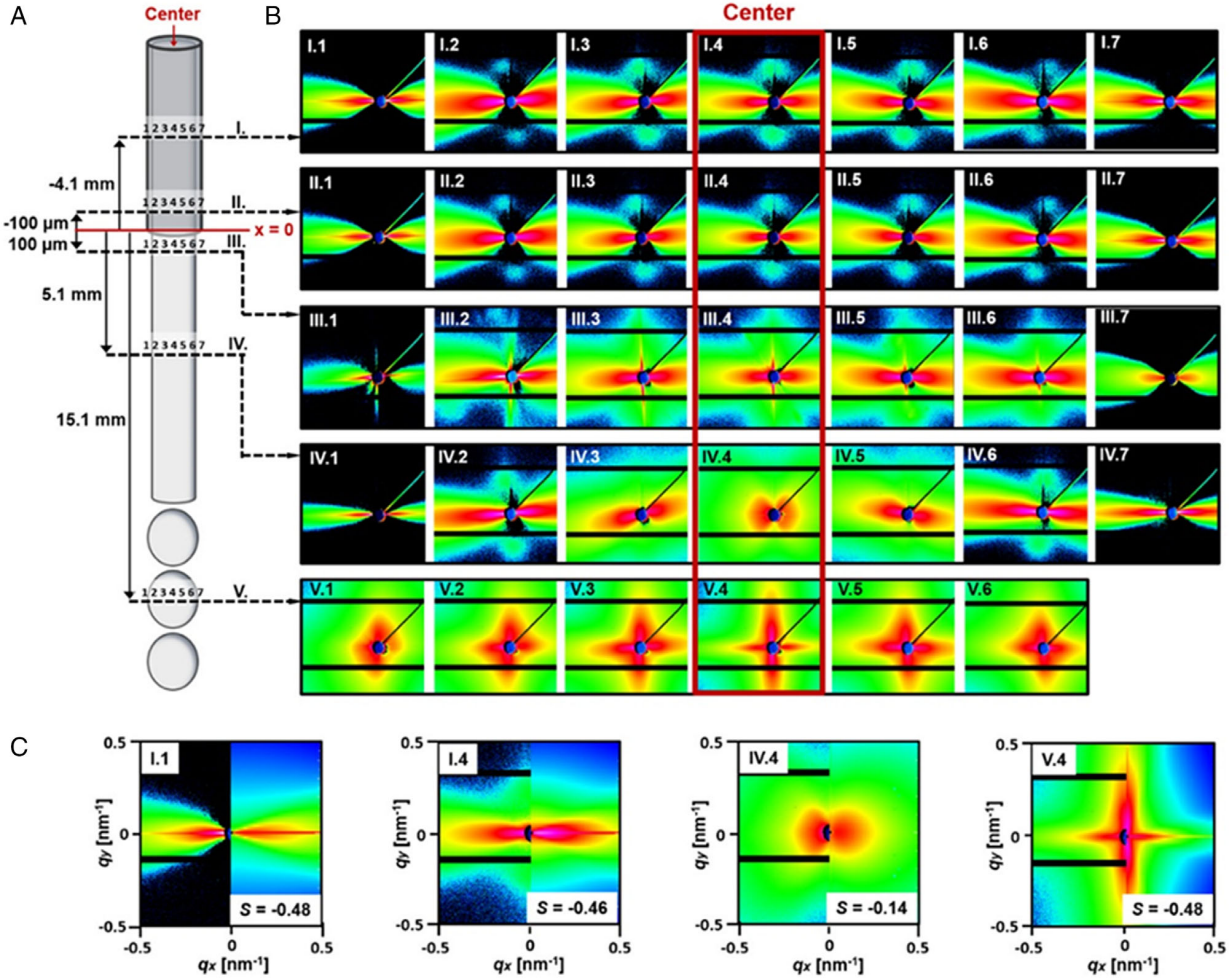
Orientation of nanoplatelets in droplets. Scheme of the microjet and the emerging microdroplets with dispersed nanoplatelets together with the 2D‐SAXS patterns measured within the jet and the droplets. (A) Scheme of the capillary, the liquid microjet and the microdroplet region with the scan lines *I−V* where the 2D‐SAXS patterns were measured. (B) Set of scattering patterns measured for the hectorite nanoplatelets at the specified scan positions. (C) Comparison of the experimental and calculated scattering patterns to determine the orientational order parameter of the nanoplatelets. The *q*‐range is −0.5 to 0.5 nm^−1^. Reproduced with permission.^[^ ^50^ ^]^ Copyright 2018, American Chemical Society.

Detailed SAXS was able to reveal that in addition to the alignment, some anisotropic nanoparticles are able to accumulate (marginate) close to the wall. In work conducted by Schlenk et al. the alignment of hectorite nanoplatelets (thickness 1.0 nm and lateral dimension of 20 µm) in stable liquid microjets were investigated using microfocus synchrotron X‐ray scattering.^[^ ^50^ ^]^ Particles were measured at different scan positions along the capillary, and strong anisotropic signals can be seen, due to the shear induced alignment of the particles (Figure 12).

Interestingly, the highest amount of scattering—shown in red‐ can be seen close to the edges of the channel, indicating a preference for platelet particles to reside close to the channel wall with their basal plane being perpendicular to the flow direction. Scattering patterns showed a slight inclination, indicating that the platelets were tilted towards the centre of the capillary. This is due to competing effects of flow orientation, with particles preferring to orient parallel to the streamlines (0° inclination angle) and rotational diffusion. This effect was also observed in wormlike micelles formed by the self‐assembly of poly(isoprene‐*b*‐ethylene oxide) (PI‐*b*‐PEO) block, but less prominent for short gold nanorods, due their fast rotational diffusion.

The ability of nanoparticles to marginate to the channel walls under laminar flow was subject to a theoretical study by Lee et al.^[^ ^51^ ^]^ At low shear rates (<100 s^−1^) margination was absent unless external forces or attractive forces with the cell wall were applied. However, at high shear rates, particles with high Stokes numbers can drift towards the wall, which explains why larger and denser particles have a larger propensity to marginate. In this theoretical study, discoidal particles with low aspect ratio displayed the highest migration towards the channel walls. Margination in pure water in clean channels is not frequently reported. However, margination towards channel wall is widely observed when the nanoparticles can interact with biological entities such as cells or protein that line the channel wall or if the nanoparticles are pushed towards the wall by red blood cells (RBC). This is the subject of the next chapter in this review.

## 4 Nanoparticles in blood flow

Blood flow can be treated in a similar way to the flow of nanoparticles in confined spaces. Blood components such as blood cells or proteins can be considered micro‐ and nanoparticles that often have anisotropic shapes. Depending on the channel width (blood vessel size), the blood pressure and the blood flow, the particles in the blood will respond to the flow profile and potentially align themselves. In this setting, we will introduce synthetic nanoparticles and identify their flow in relation to these blood components. This requires the use of additional analytical techniques that can help to distinguish between the drug carrier and nature's particles.

### 4.1 Blood composition

**FIGURE 13:**
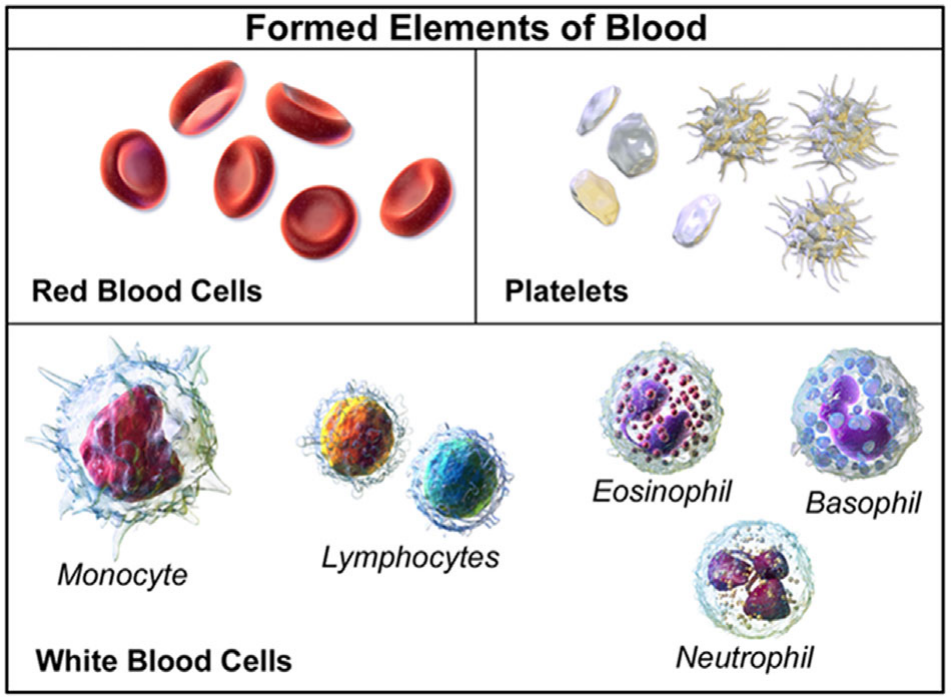
Blood components. Various cells found in blood. Repeoduced under the term of the Creative Commons CC BY‐SA 4.0 license.^[^ ^52^ ^]^ Copyright 2014, The Authors, Blausen Medical.

Blood is primarily constituted of erythrocytes, leukocytes and platelets suspended in plasma. Erythrocytes or red blood cells (RBCs) make up between 40–45% of blood by volume and are characterized by its biconcave shape ≈7–8 µm in diameter with an indented centre. Their primary function is to facilitate gas exchange though the attachment of oxygen to the haemoglobin proteins found inside the cytosol. Leukocytes or white blood cells (WBCs) which consist of granulocytes, lymphocytes and monocytes make up ≈0.1% of blood by volume and include several distinct varieties of cells, which all have different roles and structures which mediate the immune response. These cells are outlined in Figure 13.

Lymphocytes, for example, are involved in adaptive immunity and produce antibodies to fight pathogens. This is achieved by continually flowing through the circulatory system, near the blood vessel walls in order to adhere and contribute to the immune response at an inflammation site. Thrombocytes or platelets are cell fragments which are roughly disc‐shaped and about 2–4 µm in diameter that are responsible for coagulation at wound sites, initiation a signalling cascade for a wider immune response.^[^ ^53^ ^]^ Similarly, to WBCs, platelets need to be close to the blood vessel wall in order to initiate coagulation and are found to laterally flow towards the vessel walls, which is associated with their shape, size, stiffness as well as the hemodynamic conditions in flow.^[^ ^54^ ^]^ Plasma is mainly comprised of water (90%) as well as various proteins, ions and gasses and has Newtonian viscous properties.^[^ ^55^ ^]^

### 4.2 Microvasculature architecture

**FIGURE 14:**
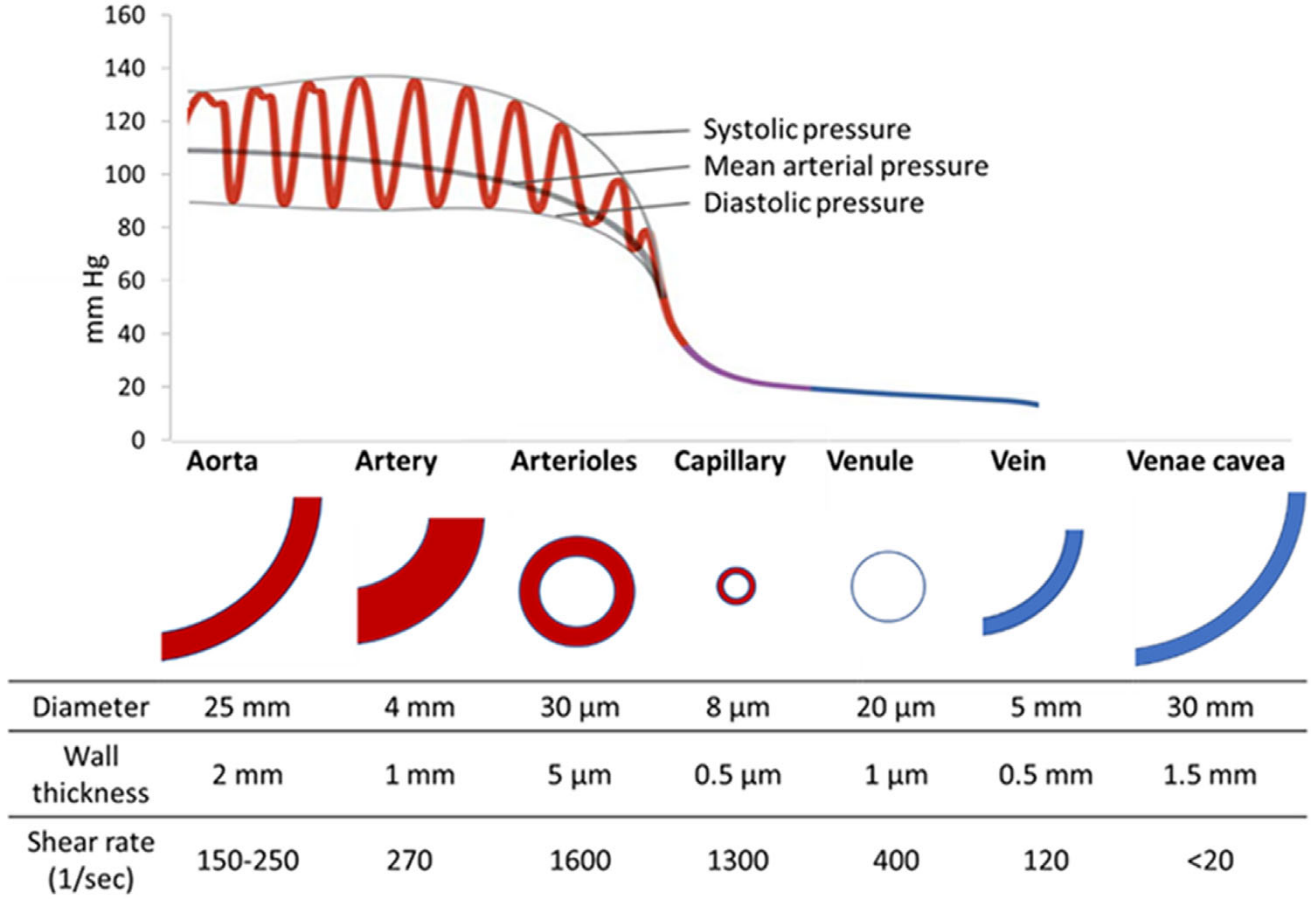
Blood vasculature. Physical properties of vasculature vessels showing the blood pressure profile in relation to the various vessel types.

The microvasculature consists of three main vessels: arterioles, venules and capillaries.^[^ ^56^ ^]^ Arterioles transport oxygenated blood to the capillaries, where it can then perfuse into the tissue before flowing out of the capillaries through venules into veins. Arterioles range from 10–100 µm in diameter and form a complex fractal branching networking. Structurally, arterioles consist of and inner endothelial cell layer laying on top of a basal membrane, with a smooth muscle layer beneath.^[^ ^40^ ^]^ This muscle layer is innervated, meaning vasoconstriction and vasodilation can occur and allows the regulation of blood pressure. Vessel diameters and compositions are outlined in Figure 14.

Capillaries are the smallest vessels ranging from 5–8 µm in diameter. Their primary role is to facilitate gas exchange between the blood and surrounding tissue by minimizing diffusion distance and maximizing surface area. Capillary walls consist of a monolayer of endothelial cells, coated in a glycocalyx surface layer which plays an important role in regulating hemodynamic resistance, and lacks the smooth muscle layer of other vessels, preventing regulation of blood pressure.^[^ ^57^ ^]^

Post‐capillary venules collect the blood from several capillaries and transport the blood into veins to be recycled and reoxygenated. The venule network mirrors the arteriolar network in structure, with the venous network being more extensive and having thinner walls when compared to its counterpart. Constant blood flow in larger micro vessels like arterioles and venules is laminar, and due to the distance from the heart, is not impacted by the pulsatile flow regime found in vessels closer to the heart.

### 4.3 Hemodynamics in the microvasculature

Hemodynamics describes the dynamic of blood flow. The complex nature of microcirculation can be simplified and modelled using Poiseuille's law, which describes the behaviour of a Newtonian fluid in laminar flow.^[^ ^58^ ^]^

$$Q=\frac{\pi r^{4}}{8l\eta }\;\Delta P$$

This equation works on the assumption that:

(1) The flow is laminar through a pipe of constant, circular cross‐section.
(2) The fluid is incompressible and Newtonian.
(3) There is no acceleration of fluids in a pipe.

where *Q* is the volumetric flow, *r* is the radius of a tube, *l* is its length, η is the dynamic viscosity of the fluid and ΔP is the pressure difference. While blood flow in larger micro vessels like arterioles and venules is laminar, these other conditions are usually not met, making Poiseuille's law a good approximation but not completely descriptive of the behaviour of blood. For example, Poiseuille's law assumes an average shear stress value that is constant along the arterial tree, whereas in vivo studies have shown vast differences in wall shear stress values along the arterial tree.^[^ ^59^ ^]^

The hemodynamic forces present in the body arise mostly from the frictional force of blood flowing against the vessel walls. In arterioles and veins, the flow profile is generally laminar and high wall shear stress (WSS) is observed, with arterioles having the highest WSS. Typical WSS in the body is dependent on the vessel and ranges from 0.5–60 dyn cm^‐^^2^ (Figure 14).**^[^** ^60^ **^]^**

Fahraeus effect describes the decrease in concentration of RBCs as the diameter of the vessel it is flowing in decreases. This effect originates from the hydrodynamic interactions between deformable RBCs interacting the endothelial wall of the blood vessel, causing a lateral migration of cells toward the centre of the channel. This migration of cells causes the blood to separate into two distinct phases: the viscous RBC phase in the centre of the channel and the cell depleted phase close to the vessel wall known as the cell free layer (CFL). Another consequence of the formation of the CFL is the decrease in apparent blood viscosity in vessels larger than 7 µm and a drastic increase in viscosity in vessels smaller than 7 µm.^[^ ^24e^ ^]^

## 5 UNDERSTANDING PARTICLE MARGINATION IN THE BLOOD

### 5.1 Margination in the body

**FIGURE 15:**
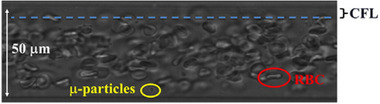
Cell‐free layer (CFL) formation in a capillary. The occurrence of a cell free layer close to the vessel wall where no RBC, but sometimes nanoparticles can be found. Reproduced with permission.^[^ ^29^ ^]^ Copyright 2015, Elsevier.

Margination in blood is a phenomenon characterized by the movement of particles or cells in blood towards the vessel walls. Although margination can be observed under specific circumstances in blood‐free medium in small channels, it is more pronounced in blood. This occurs primarily in the microvasculature, where blood does not behave like a Newtonian fluid. In blood vessels with diameters less than 500 µm, red blood cells (RBCs) move towards the center of the vessel due to wall‐induced hydrodynamic lift.^[^ ^54^, ^57^, ^61^ ^]^ This is due to the deformable nature of RBCs, which collide with vessel walls creating an asymmetric pressure field, pushing the RBCs away from the wall, which in turn pushes other RBCs away from the wall. As a result, the area close to the vessel walls contains very few RBCs (Cell‐free layer: CFL) (Figure 15).

Margination occurs when a particle, most commonly white blood cells and platelets in the body, which also tend to be more rigid and less deformable than RBCs, collide with RBCs, resulting in shear‐induced diffusion of these particles towards the CFL. The ability of a particle to marginate is dependent on several blood related factors and particle attributes. Vessel diameter, wall shear stress, haematocrit and shape all influence margination.^[^ ^61^, ^62^ ^]^ Similarly, particle size, shape, density and stiffness are also important factors to consider. For example, stiff particles tend to accumulate in the centre while harder particles migrate to the walls.^[^ ^63^ ^]^ The ability to synthesize particles with optimal margination provides another design consideration to researchers in particle‐based drug delivery because the proximity to the vessel walls allows these particles greater chance for adhesion to the endothelium and thus more opportunities to leave the circulatory system. Increasing particle margination offers a way to prolong the circulation of particles within the body as well as an avenue for more effective delivery of therapeutics. This is particularly attractive to researchers in the field of drug delivery for tumours, as tumour vasculature is characteristically leaky, with large fenestrations that are 200 nm wide present along the endothelial wall.^[^ ^24b^ ^]^

### 5.2 Computational studies on margination

A range of models, reviewed earlier by Kumar et al., have been developed to help understand the segregation of two and more particles in flow, including the segregation of RBC, other blood components like platelets and nanoparticles and.^[^ ^63^ ^]^ In work conducted by Chang et al., simulations were used to quantify platelet margination in diabetic blood flow.^[^ ^62d^ ^]^ Detailed simulations of RBCs in the microvasculature were used to determine the percentage of platelets found close to the vessel wall. A range of physiologically relevant wall shear rates at different haematocrit values were measured. Increases in wall shear rate and haematocrit values both lead to an increase in margination. Interestingly, an aspect ratio of 0.38 was found to be the optimal shape, outperforming aspect ratios of 0.28 and 0.5. For low aspect ratio particles of 0.28, less margination was seen as a function of decreasing haematocrit, whereas the flatter 0.5 aspect ratio particles were less affected by this change. In another computational study, Qin et al. examined the mechanisms governing margination with variation in channel height, shear rate and haematocrit value (Figure 1).^[^ ^64^ ^]^

Simulations found that the cell‐free layer increases weakly with channel height, and thus time for CFL formation increases with increasing height. Particle margination was found to significantly increase with higher haematocrit, a finding also reported in simulations by Katanov et al.^[^ ^65^ ^]^ At lower haematocrit, simulations indicated that platelets are less likely to be located near the channel wall, being more dispersed over a wider CFL. Platelet margination was found to primarily be governed by RBC‐platelet interactions, although complementary platelet‐platelet interactions exist within the CFL. The boundary between the CFL and bulk RBC creates a drift force, which forces platelets into the CFL. However, this force does not affect platelets in the centre of the RBC rich region, which undergo a much slower margination process through shear‐induced diffusion.

Similar to platelets naturally found in blood, the movement of nanoparticles is affected by RBC in similar ways. Using a theoretical model that is based on hemodynamic forces such as blood viscosity and shear rate, nanoparticle buoyancy in blood and forces between particles, Decuzzi et al.^[^ ^66^ ^]^ predicted that the maximum margination time, the time needed to reach the wall, can be found at intermediate particles sizes at around 100 nm Yet, this number is influenced by the density and steric stabilization of the nanoparticle.

### 5.3 In vitro studies on margination

In order to determine if margination is occurring, appropriate microvascular mimicking systems have to be synthesized as outlined earlier in this article. The most frequent way this is achieved is by using PDMS microchannels, which allows great customizability regarding the dimensions of the channel. Once a channel of appropriate size (usually 20–200 µm in width) is generated, RBCs and particles can be flowed through the device to mimic blood vessel behaviour. Choosing biologically relevant shear rates and haematocrit are key here, so that results generated are based on the blood vessel shear rates and haematocrit you wish to emulate. Choice of RBC can also be important, with bovine and equine RBCs being adequate (and safer) substitutes to human RBCs.

Typically, margination is observed by labelling the particle of interest with a fluorescent dye and then using a combination of fluorescence and brightfield microscopy.^[^ ^17^, ^29^, ^61^, ^67^ ^]^ Fluorescence microscopy allows particles to be seen clearly through RBCs, which is not possible with just brightfield microscopy. This is required to precisely track particle positions accurately and to confidently differentiate particles from RBCs. While polarized light microscopy and SAXS are frequently used to track particles in flow, the high light and X‐ray scattering of RBCs makes utilizing these techniques to track particle margination difficult. However, a recent study using polymer platelet was able to use fluorescence microscopy and SAXS analysis to reveal the margination of these 2D structures. The platelets were labelled with a fluorophore to enable analysis with the help of fluorescence microscopy while attachment of gold nanoparticle increased the contrast between synthetic material and RBC to such an extent that it was possible to obtain SAXS signals across the channel in a complementary manner.^[^ ^68^ ^]^

Margination can be quantified in several different ways, however in work conducted by Carboni et al., the velocity of particles tracked were normalized relative to their *y*‐axis position in a rectangular microfluidic device.^[^ ^61^, ^67^ ^]^ Fluorescently labelled particles were flowed through microfluidic channels at different wall shear rates, and their position relative to the channel wall was recorded using fluorescence microscopy. By weighting particles found in the centre of the channel less than those found at the channel wall, due to the velocity of the particles in the centre channel being far higher than at the walls and thus particles in the centre of the channel will be overrepresented during imaging.

$$P_{i^{th}\;segment\in \left[1,10\right]}=\sum_{j\in \left[1,n\right]}\frac{v_{x,max}}{v_{x,j}}$$

A margination parameter *M* can then be calculated by adding the number of particles found in the 1st and 10th segment of the microfluidic device and dividing that by the sum of all particles observed. With this parameter a number greater than 0.2 indicates margination, as a system with no margination would expect 20% of the particles to be found at the channel walls.

$$M=\frac{P_{1^{st}\;segment}+P_{10^{th}\;segment\;}}{\sum_{i^{th}\;segment\in \left[1,10\right]}P_{i}}$$

With a way to quantify margination with the parameter *M*, the effect of size on margination was investigated, using 0.53, 0.87 and 2.11 µm latex spheres under different shear rates. Measurements were conducted using a 100 µm wide PDMS channel, measuring 10 mm in length. Bovine RBCs were used in place of human RBCs and were washed and adjusted to a haematocrit value of 35%. The results showed that the largest 2.11 µm spheres had the largest margination parameter *M* of 0.3, measured at a shear rate of 61 s^−1^. The smaller spheres showed little to no margination, with M values lower than 0.2, indicating no preference for accumulation near the channel walls.^[^ ^61^, ^67^ ^]^ Using this approach, a significant degree of margination can be observed when using polymer platelets with length scales of around 1 and 2 µm. The smaller platelet was seen to have *M* values of almost 0.4 at high blood flow rate.^[^ ^68^ ^]^ Margination in the presence of RBC was observed for a range of systems such as spherical polymer nanoparticles of 10 and 100 nm,^[^ ^69^ ^]^ micron‐sized polymer platelets prepared by crystallization induced self‐assembly,^[^ ^68^ ^]^ SiO_2_ discoidal particles,^[^ ^62b^ ^]^ PS spheres with targeting ligand sialyl LewisA,^[^ ^62^, ^70^ ^]^ and PEG‐coated nanoparticles^[^ ^71^ ^]^ (Table 1) In general, margination in the presence of RBC was more prominent in larger particles and non‐spherical shapes, especially disks.

**TABLE 1:** Summary of important flow experiments using spherical and non‐spherical nanoparticles in channels coated with protein or cells or in blood.

Nanoparticles	Particle size	Channel type	medium	Flow rate (µm min^‐1^) or shear rate (s^−1^)	Outcomes	Ref
Block copolymer micelle with different surface chemistries	40–130 nm	HUVEC coated	Aqueous solution	1.0 µm min^−1^	Reduced adhesion in flow compared to static, size correlation is dependent on surface charge	^[^ ^31^ ^]^
Liposomes and micelles with different sizes and surface charges	40–150 nm	HUVEC coated	Whole blood	5.0 µm min^−1^	Cellular association decreases with size, strong tendency to be taken up by B‐cells instead	^[^ ^75^ ^]^
SiO_2_: microspheres, discoidal quasihemisperical	1 µm (spherical) 1.5 µm × 0.3 µm (disc) 3.2 µm (quasih.)	collagen coated	Aqueous solution	32 to 322 µm min^−1^	More margination in discoidal particles	^[^ ^62b^ ^]^
polystyrene spheres coated with sialyl lewisA: spherical	200 nm, 500 nm, 2 µm, and 5 µm	HUVEC coated	Red blood cells	100, 200, and 500 s^−^^1^	Larger spheres have higher margination and better adhesion to cells	^[^ ^62e^ ^]^
polystyrene spheres coated with sialyl lewisA: spherical	100 nm to 10 µm	HUVEC coated	Red blood cells	Laminar (200 s^−1^) and pulsatile flow (1000 s^−1)^	Adhesion and margination increased with size, concentration; pulsatile flow enhance effect for large particles only	^[^ ^70^ ^]^
Particles of different densities, spheres and rods (liposomes and metal), density ranging from 1 to 19.3 g mL^‐1^	56–130 nm	coated with fibronectin	Aqueous solution	50 µm min^‐1^	Smaller nanoparticles and particle with lower density, adhere quicker to wall	^[^ ^76^ ^]^
Fluoresbrite Microspheres	50 nm, 100 nm, 200 nm, 500 nm, 750 nm, and 1 µm, 6 µm, 10 µm	HUVEC coated	Aqueous solution	50 µm min^‐1^	Increased margination with larger particles	^[^ ^77^ ^]^
Spheres and rod using mathematical model	100 and 200 nm (rods) Rod with aspect ratio of 3 and 5	Mathematical model of channel with bifurcation	–	Modelling using 100, 200, 400, 600, and 1,000 s^−1^	Less binding to wall with increased shear rate, faster binding of smaller particles, no better binding by rods	^[^ ^78^ ^]^
Spherical PEG coated polystyrene	200 and 1000 nm	In vivo	blood	In vivo	Larger nanoparticles accumulate near wall while smaller move with RBC	^[^ ^71^ ^]^
spheres, elliptical/circular disks and rods, coated with anti‐BSA	1–20 µm	BSA coated	Aqueous solution	15 to 250 s^−1^	Large rods and circular disk exhibit highest adhesion on channel wall	^[^ ^28c^ ^]^
Anti‐ICAM‐1 coated spherical micro/nano particles	210 nm and 2 µm	intercellular adhesion molecule 1 (ICAM‐1) protein coated	Aqueous solution and whole blood	200 and 1600 s^−1^	Larger particles attached better than small particles in the presence of blood; without blood attachment is dependent on shear rate	^[^ ^28b^ ^]^
Poly(lactic‐*co*‐glycolic acid) (PLGA) spheres, discs and rods	0.9 and 3.0 µm (spheres), 1 µm × 400 nm (discs), 400 nm × 1.8 µm (rods)	Non coated channels and in vivo	Albumin solution and whole blood	20 mm Hg	RBC trigger margination in small spheres; higher margination in bigger particles, rods show lowest margination; in vivo confirms flow results	^[^ ^29^ ^]^
polystyrene spheres	2.11 µm	Non‐coated with constrictions	blood	0.6 µL min^‐1^	No margination without blood, margination increased in constriction	^[^ ^61a^ ^]^
Polystyrene rods coated with sialyl lewisA	0.52–8.97 µm length, different aspect ratios	HUVEC coated	Red blood cells	Laminar (200 s^−1^) and pulsatile flow (1000 s^−1)^	Adhesion of rods only improved compared to spheres at specific rod lengths	^[^ ^62a^ ^]^
polystyrene spheres	0.53, 0.84, and 2.11 µm	Non‐coated	Aqueous solution and blood	30, 61, and 121 s^−1^	More margination with higher shear rate, in blood and with larger sphere sizes	^[^ ^67^ ^]^
Polymer platelets prepared crystallisation‐driven assembly	1 and 2 µm × 20 nm	Non‐coated	Red blood cells	50, 100, and 200 s^−1^	Highest margination with 1 µm platelets	^[^ ^68^ ^]^

### 5.4 In vivo studies on margination

**FIGURE 16:**
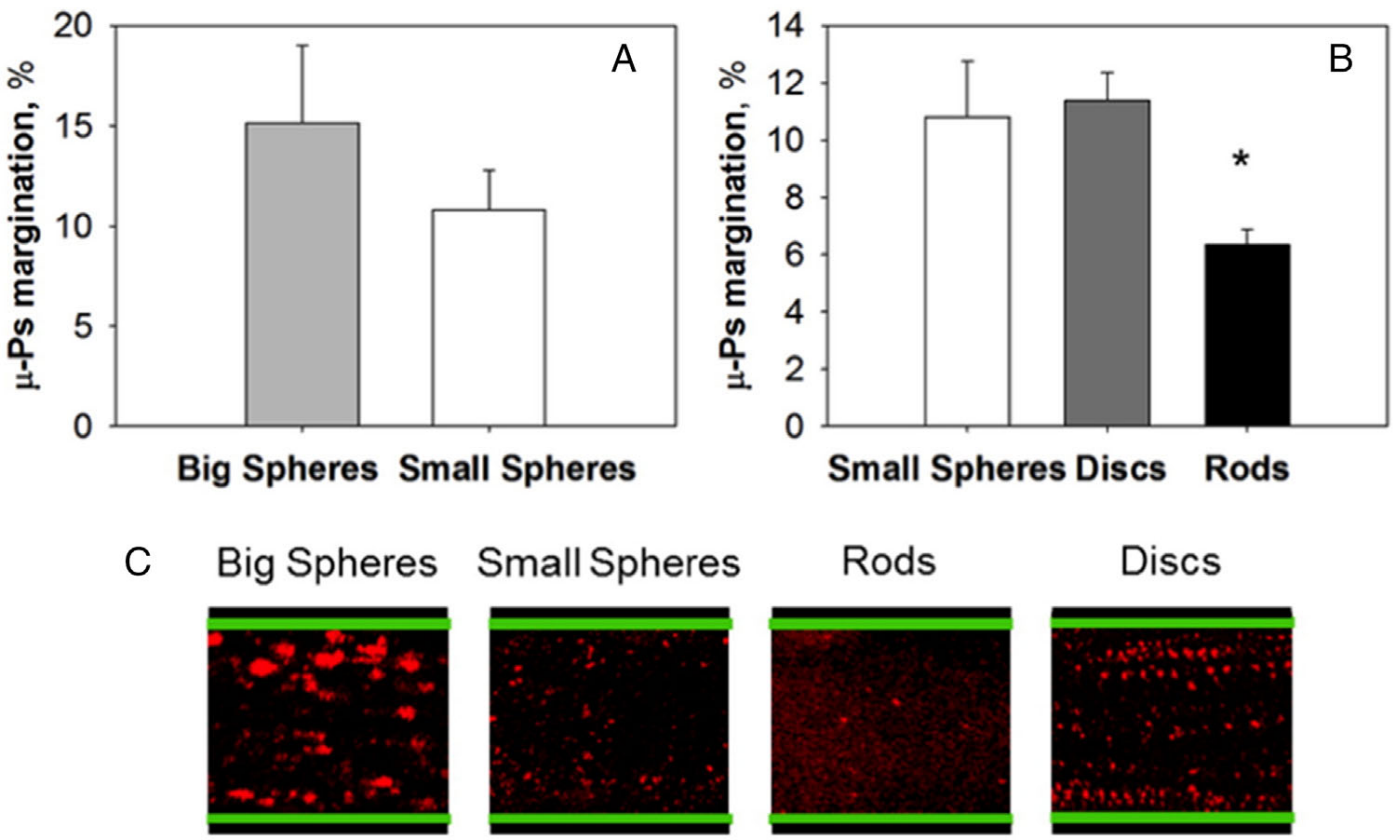
Margination of particles with different shapes. Percentage of marginated µ‐Ps in the presence of RBCs (Hct 10%) at 20 mm Hg by intravital microscopy. (A) Comparison between particles with the same shape and different sizes: the difference between small and big spheres is not significant. (B) Comparison among particles with the same volume and different shapes. (C) Dispersion map obtained merging the time frames of a movie acquired using intravital microscopy while µ‐Ps were flowing into the vessels (capillary walls are shown in green and µ‐Ps in red). Reproduced with permission.^[^ ^29^ ^]^ Copyright 2015, Elsevier.

In vivo margination experimental have been carried out using spherical nanoparticles where it was shown that nanoparticles of 1000 nm are more likely to marginate to the blood vessel compared to smaller (200 nm) nanoparticles.^[^ ^71^ ^]^ As the topic of this article is the flow of non‐spherical particles, we would like to discuss however in more detail studies that have been conducted by D'Apolito et al., where the margination properties of spheres, rods and discoidal particles were compared between microfluidic capillaries as well as in vivo blood vessel characterization in genetically modified mice (Figure 16).^[^ ^29^ ^]^

Poly(lactic‐*co*‐glycolic acid) (PLGA) microparticles were synthesized using micropatterning and cast into small spheres (1 µm diameter), large spheres (2 µm diameter), rods (1.8 µm *L*_n major,_ 400 nm *L*_n minor_) and discoidal particles (1.8 µm diameter, 400 nm thickness). These particles were then studied in vivo, using intravital confocal fluorescence microscopy, by fluorescently tagging microparticles with rhodamine, and tracking their position relative to the vessel walls, which have been genetically modified to express green fluorescence protein. The animals were placed under a microscope and the vessels close to the skin were exposed by a small incision. Margination was quantified not by using the Margination parameter *M*, but by plotting the percentage of particles found as a function of the distance from the centre of the capillary. Results showed 25% of large 3 µm spheres were found in the CFL compared to ≈18% for smaller 1 µm spheres and discoidal particles, with rodlike particles demonstrating the least amount of margination with less than 13% found in the CFL. This was consistent with previous in vitro results that were obtained using the same particles under similar conditions. The ability of spherical and discoidal particles to remain close to the endothelial wall is thought to arise from the higher frequency of collisions with RBCs. Since disks exhibit both flipping and tumbling motion around their longitudinal axis, there was a higher probability to interact with RBCs and move into the CFL. However, rod‐like particles, which align with their major axis along the capillary axis, revolve primarily along the axis perpendicular to the longitudinal axis, resulting in fewer collisions with RBCs. In vitro studies, which involved measuring the number of particles found close to the CFL using a 50 µm wide silica capillary at 20 mmHg and tracking particle position using high speed microscopy. Results from this experiment showed that trends seen in vitro were consistent with those performed in vivo.

## 6 MEASURING PARTICLE ADHESION

### 6.1 Adhesion studies

**FIGURE 17:**
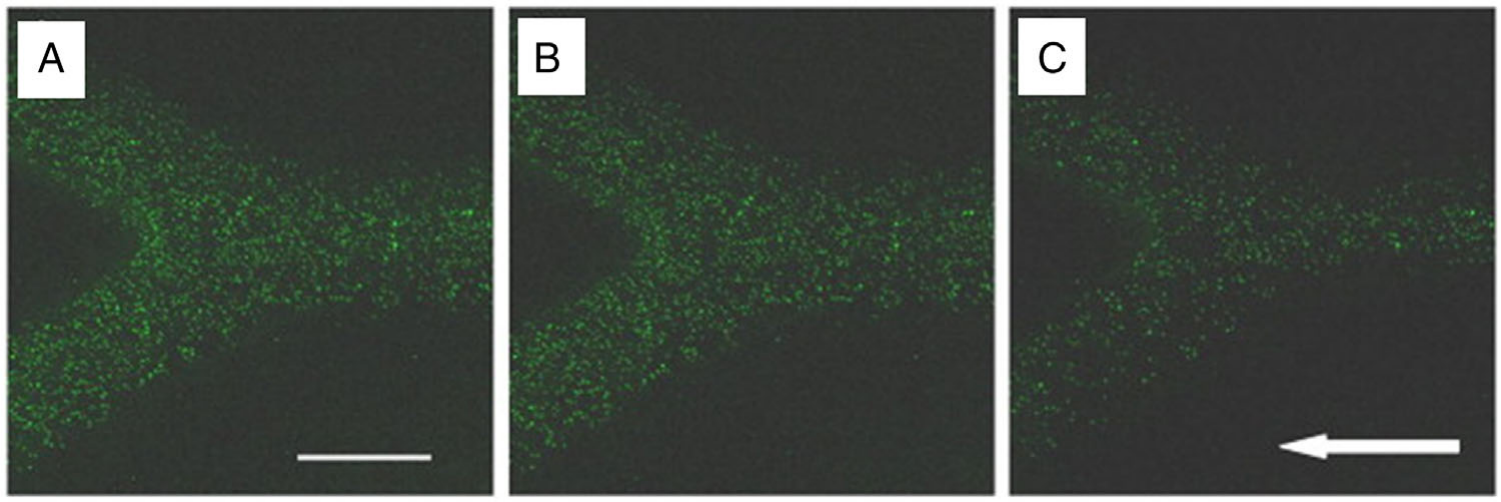
Adhesion of ligand coated nanoparticles. Fluorescently labelled 210 nm PS particles with anti‐ICAM 1 adhered in ICAM‐1 coated branched channel using three anti‐ICAM‐1 particle coating densities: (A) 1905.3 µm^−2^; (B) 1257.1 µm^−2^ and (C) 625.8 µm^−2^. Reproduced with permission.^[^ ^28b^ ^]^ Copyright 2014, Elsevier.

**FIGURE 18:**
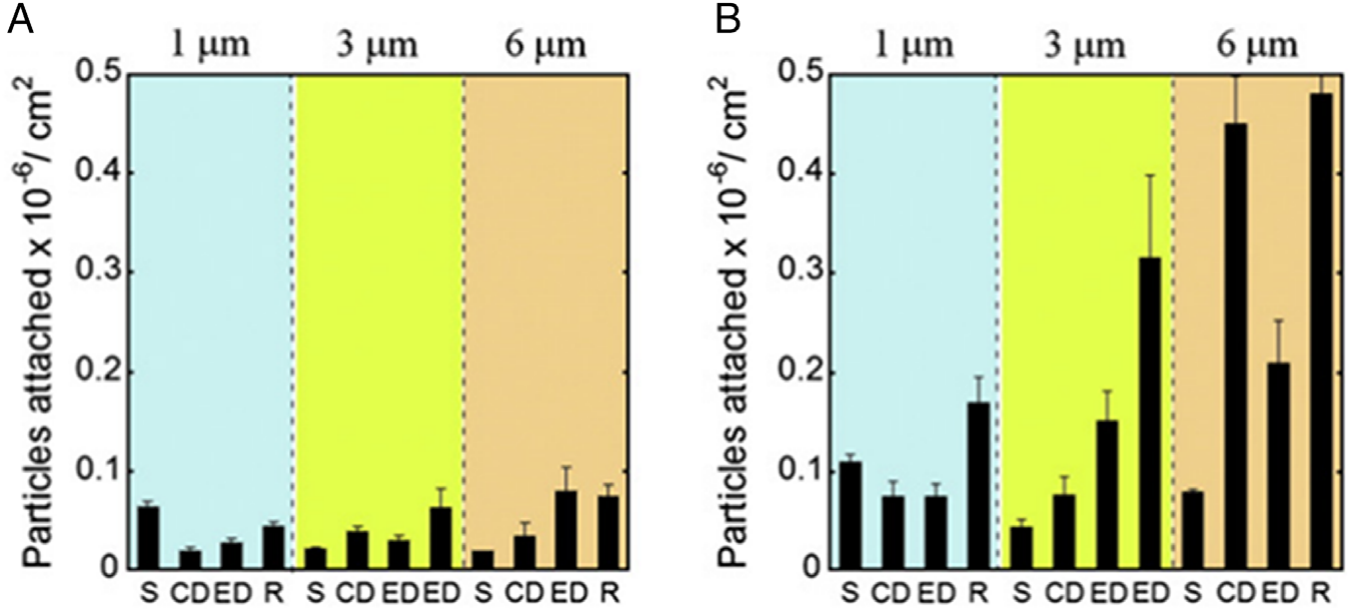
Particle attachment anti‐BSA coated nanoparticles to BSA coated channels. Comparison of the adhesion propensity of carriers in terms of the number of particles attached per unit area for all shapes and sizes used for the study at the lowest shear rate of 15 s−1. (S = spheres, ED = elliptical disks, CD = circular disk, R = rods. For 3 µm particles, the two EDs correspond to low aspect ratio and high aspect ratio elliptical disks). (A) Particle attachment profile at the inlet section. (B) Particle attachment profile at the junction. Reproduced with permission.^[^ ^28c^ ^]^ Copyright 2010, Elsevier.

While understanding particle behaviour in flow is important, understanding the ability of particles to attach to the endothelial wall becomes the next important question in the journey of a particle in the body. Attachment to the endothelial wall is the first step to a particle leaving the circulatory system and arriving at its desired site. This phenomenon can be measured by the adhesion of a particle to a channel surface in flow. Adhesion is dependent on a variety of factors, including, surface chemistry, shape, particle density and size (Table 1).^[^ ^28^, ^72^ ^]^ For spherical particles, adhesion is determined at a discrete shear stress by its diameter.^[^ ^73^ ^]^ Also denser particles are usually favourable when trying to achieve enhanced interaction with the vascular system.^[^ ^74^ ^]^ However, for non‐spherical particles, no such simple correlation exists and determining adhesion is found by measuring the number of particles bound to a channel surface (Figure 17).^[^ ^28b^ ^]^ This is frequently achieved by coating the walls of a PDMS microfluidic device with a protein or antibody, and then attaching the complementary protein to the particle you wish to study.^[^ ^28b,c^ ^]^ The degree of adhesion is then measured by calculating the binding density, which is the number of particles adhered to the surface of a microfluidic channel per mm^2^ of the channel outlined in Figure 18.

This provides a quantitative method of determining the effectiveness of particle binding, with the influence of shape, size and flowrate all important parameters able to be measured. For instance, a bifurcated PDMS microchannel was coated with the intercellular adhesion molecule‐1 (ICAM‐1) protein and while the particles are decorated with anti‐ICAM‐1 antibody. ELISA assays were carried out on the microfluidic channels, and particle binding density was shown to decrease with increasing shear rates for both 210 nm and 2 µm polystyrene spheres. While in buffer smaller particles adhere preferably, this effect was reversed at low shear rates in a solution with RBC, suggesting high margination of larger particles in the presence of blood. This was not true for these larger 2 µm spheres at higher shear rates, where shear‐based detachment drag force nullifies increased binding interactions.^[^ ^28b^ ^]^

With the aim of mimicking blood vessels, these channels are often coated with cells, predominantly HUVEC cells (Table 1). The HUVEC coated channel wall can now serve as an attractive force for nanoparticles as the nanoparticles tend to engage with the cell surface once in contact. This is in particular the case when targeting ligands such as sialyl lewisA are used (Table 1). It is now possible to simply count the number of nanoparticles at different locations along the channel after the cells were exposed to the circulation nanoparticle solution. When the channels are seeded with cells, it is possible to not only to measure adhesion, but also cellular uptake. HUVEC coated channels were exposed to micelles with different surface functionalities at flow rates at 1.0 µm min^‐1^ to compare the amount of cellular uptake under flow to that under static conditions. Not only was the cellular uptake under flow reduced, but the behaviour was also dependent on the surface chemistry of these spherical particles.^[^ ^31^ ^]^ Additional information was obtained when nanoparticles were mixed with blood cells and injected into these HUVEC coated channels. Liposomes and micelles showed high association with B cells under both flow and static conditions independent of the surface charge.^[^ ^75^ ^]^

### 6.2 Effect of particle shape and size on adhesion

The effect of particle shape and size on adhesion has also been investigated by measuring the number of adhered particles on a bifurcated channel.^[^ ^28c^ ^]^ Polystyrene spheres, circular discs, elliptical discs and rods of 1, 3 and 6 µm were all fabricated and coated in anti‐BSA antibodies to measure their adhesion to BSA coated channel. Significantly more adhesion was seen at the junction point than in the linear channel, with the effect of shape amplified at larger particle sizes. Anisotropic particles such as polystyrene based rods and circular disks showed the highest propensity for attachment at the junction, most likely due to the greater binding interactions they can have when oriented parallel to the channel wall, overcoming the larger shear detachment forces, which is not present in spheres. This indicates that larger anisotropic (>1 micron) particles might be better for targeting the endothelial wall (Figure 18).^[^ ^28c^ ^]^

In a review on the effects particle shape has on in vivo journey of nanoparticles,^[^ ^72b^ ^]^ discs have been reported to adhere two times more than spheres in straight channels. Discoidal particles also displayed 2–2.5‐fold higher ratio of adhesion in bifurcated channels, which indicates even greater binding advantages in more complex vessel geometries. Both particle size and aspect ratio were again identified as key determinants that affect the level of adhesion.

Density also has an important effect on particle binding, with denser particles carrying more momentum, causing them to travel a shorter distance after colliding with RBCs when compared to lighter particles of similar size. This requires more energy to move the denser particle and should affect its ability to adhere. In a study by Toy et al., adhesion between 65 nm diameter gold nanospheres and liposomes were compared for their ability to bind to fibronectin coated microchannels.^[^ ^72a^ ^]^ The results of the adhesion studies showed that liposomes deposited on the channel wall 57 times greater than the gold nanospheres. Even differences in the density of cargo carried by the liposome can affect its adhesion properties, with liposomes loaded with iodine showing significantly less adhesion when compared to liposomes with water encapsulated. Other results from this study indicated that smaller particle (65 nm) adhere to channel walls more than larger particles (100 nm) regardless of shape, with gold nanorods also having a much greater rate of attachment when compared to nanospheres of similar size.^[^ ^72b^ ^]^

These studies indicate that particle adhesion is influenced by both particle specific properties, such as size, density and shape, as well as flow specific conditions (Table 1). Shear rate, channel dimensions and material and presence of RBCs determine the outcome of particles in vascular like conditions. In general, microparticles have been shown to possess better margination and adhesion properties when compared to nanoparticles. Anisotropic particles demonstrate better margination and adhesion properties when compared to spheres of similar volumes, with discs marginating and adhering more than rods.^[^ ^62^, ^67^, ^72^ ^]^

## 7 CONCLUSIONS AND PERSPECTIVE

In conclusion, while the relationship between particle shape, size and density and their effectiveness in drug delivery are not clear, anisotropic particles demonstrate increased margination and adhesion properties when compared to spherical particles of comparable size. By better understanding of how particles behave in flow, including orientation, margination and adhesion properties, researchers can better predict how these particles may behave in vivo. This can be achieved through the incorporation of experimental techniques such as polarized light microscopy and SAXS in flow, which can provide interesting structural and orientational information. Additionally, use of customizable microfluidic channels which can more closely resemble conditions within the human body can help researchers understand the specific microenvironment their particles aim to deliver therapeutic payload to.

So far, the flow of anisotropic nanoparticles in pure solvents such as water or organic solvents have been widely studied. Theoretical studies as well as experimental results have shown how anisotropic nanoparticles align themselves in relation to their structure such as their length, stiffness and thickness. Essential is also the design of the channel or capillary to achieve high degrees of alignment. We have now enough tools to predict the outcomes although there are still areas that deserve exploration.

Most commonly, short straight channels have been used for these investigations. However, constriction or bifurcations can disrupt the linear alignment, and the subsequent behaviour can be unexpected as seen in the perpendicular arrangement of cylindrical polymer micelles after exciting a constriction. Investigating the flow of nanoparticles along bends, branching and uneven channel thicknesses or surfaces could be an important step towards our understanding of nanoparticles in the blood flow. After all, our vascular system consists of small and large blood vessels that are branched and uneven. Trying to understand how nanoparticles flow in such a complex system is highly challenging, but microfluidic systems can capture typical design features of vascular systems.

Even more complex than non‐linear channel designs are channels that are filled with blood instead of water. Now, the dominance of red blood cells will determine the fate of nanoparticles. Due to the presence of the scattering blood cells, it is not easily possible anymore to identify the location and orientation of nanoparticles using SAXS or light microscopy. Here, the researchers turn to fluorescently labelled nanoparticles and with the help of fluorescence microscopy, it is possible to identify the position of the nanoparticles within the channel. The extent of margination can now be identified and related to the nanoparticle structure. While a solid body of work is emerging in this area, the type of nanoparticles tested so far is limited. Considering the myriads non‐spherical nanoparticles available in literature, there are opportunities to answer the question what type of nanoparticle might have the highest margination.

The reader might have noticed that nanoparticle alignment in solvent was usually observed with light microscopy or SAXS while the flow in blood was monitored with fluorescence microscopy, although an attempt was made to combine flow in blood with SAXS. When switching fluorescence microscopy, the detailed information on the alignment and the orientation distribution are lost. The focus in this experiment is to evaluate the extend of margination, but the degree of orientation is unknown. Margination together with alignment in the blood stream are currently difficult to identify.

Although several systems have been explored in blood, there are so far limited amounts of biological evaluations that link both. It would be interesting to explore how the degree of margination will correlate to actual biological outcomes such as the circulation time, if there is a relationship at all considering the complexity of biological system. If suitable relationships are found, analysis of flow prior to animal models might help to reduce unnecessary in vivo test. However, it is currently not possible to draw any conclusions. We can however say that analysis of nanoparticles using blood‐filled microfluidic devices could be an attractive tool to study drug delivery in the case of vascular diseases such as clots. We can then take this idea a step further and incorporate cells and organs, which of course an already emerging field as organs on a chip become not common delivery platform to test drugs and also nanoparticles. However, we are now entering a whole now field, worth exploring.

## CONFLICT OF INTEREST STATEMENT

Martina Stenzel is a member of the *Exploration* editorial board. The authors declare no competing financial interest.
